# Supplementary material for: Magma flow localisation during dyke propagation produces complex magma transport pathways
Source: Nat Commun. 2025 Jul 10;16:6358. doi: 10.1038/s41467-025-61620-5 (PMC12246046; doi:10.1038/s41467-025-61620-5)
Supplement: Supplementary file 1 — Supplementary Information [file 41467_2025_61620_MOESM1_ESM.pdf]

Magma flow localisation during dyke propagation produces complex magma transport pathways

Supplementary Information

Allgood, C.[1*], Llewellin, E.W.[2*], Brown, R. J.[2], and Loisel, A.[3]

[1] *Lancaster Environment Centre, Lancaster University, Lancaster, UK*

[2] *Department of Earth Sciences, Durham University, Durham, UK*

[3] *Université de Strasbourg, CNRS, Institut Terre et Environnement de Strasbourg, Strasbourg, France*

[*] *Corresponding authors (c.allgood1@lancaster.ac.uk, ed.llewellin@durham.ac.uk)*

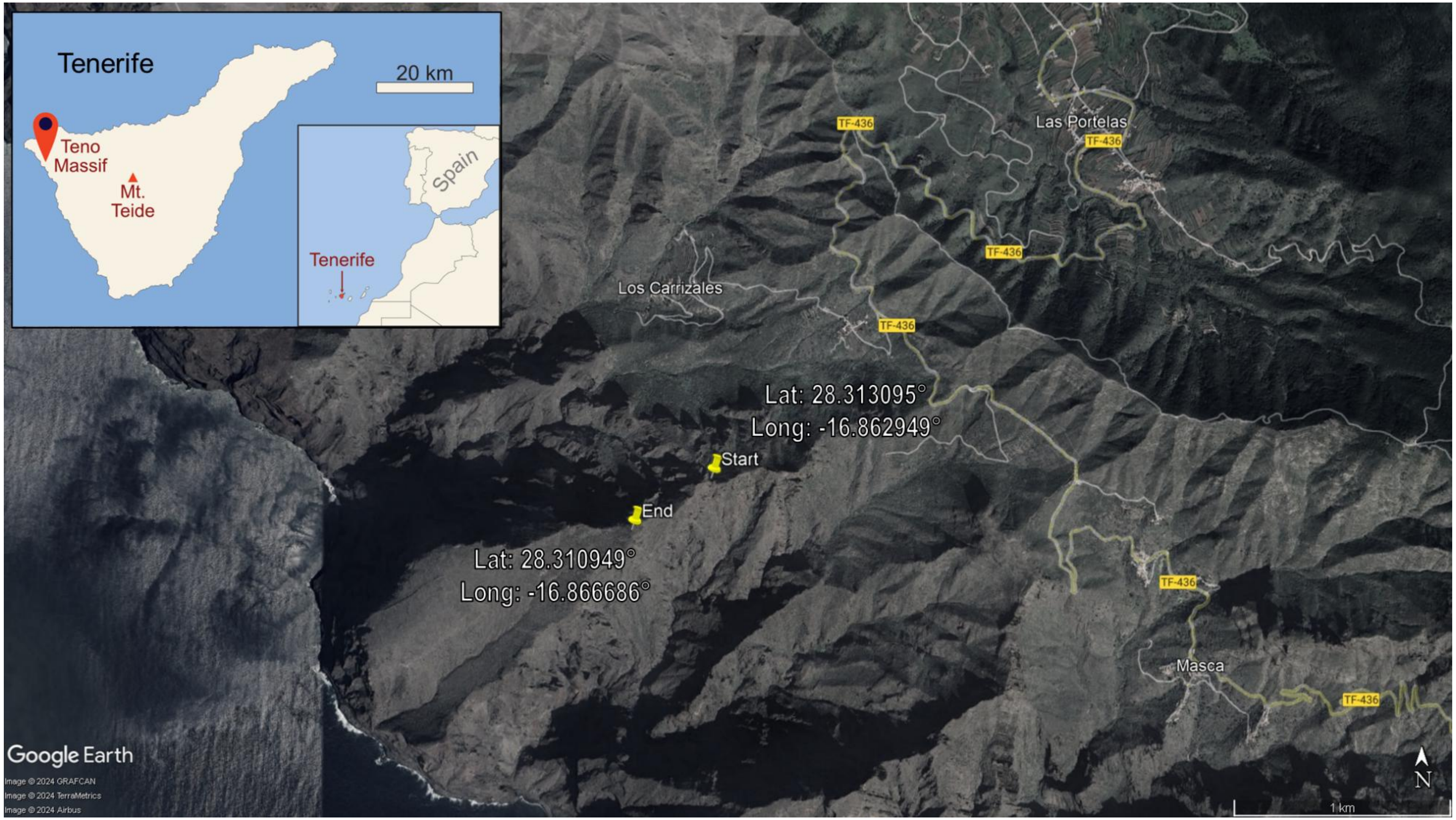

Supplementary Figure 1a. Map of study area, showing start and end location of studied dyke section. The inset shows the location of the Teno Massif on the island of Tenerife, Spain, and the location of Tenerife off the coast of northwest Africa. Map data: Google, GRAFCAN, TerraMetrics, Airbus.

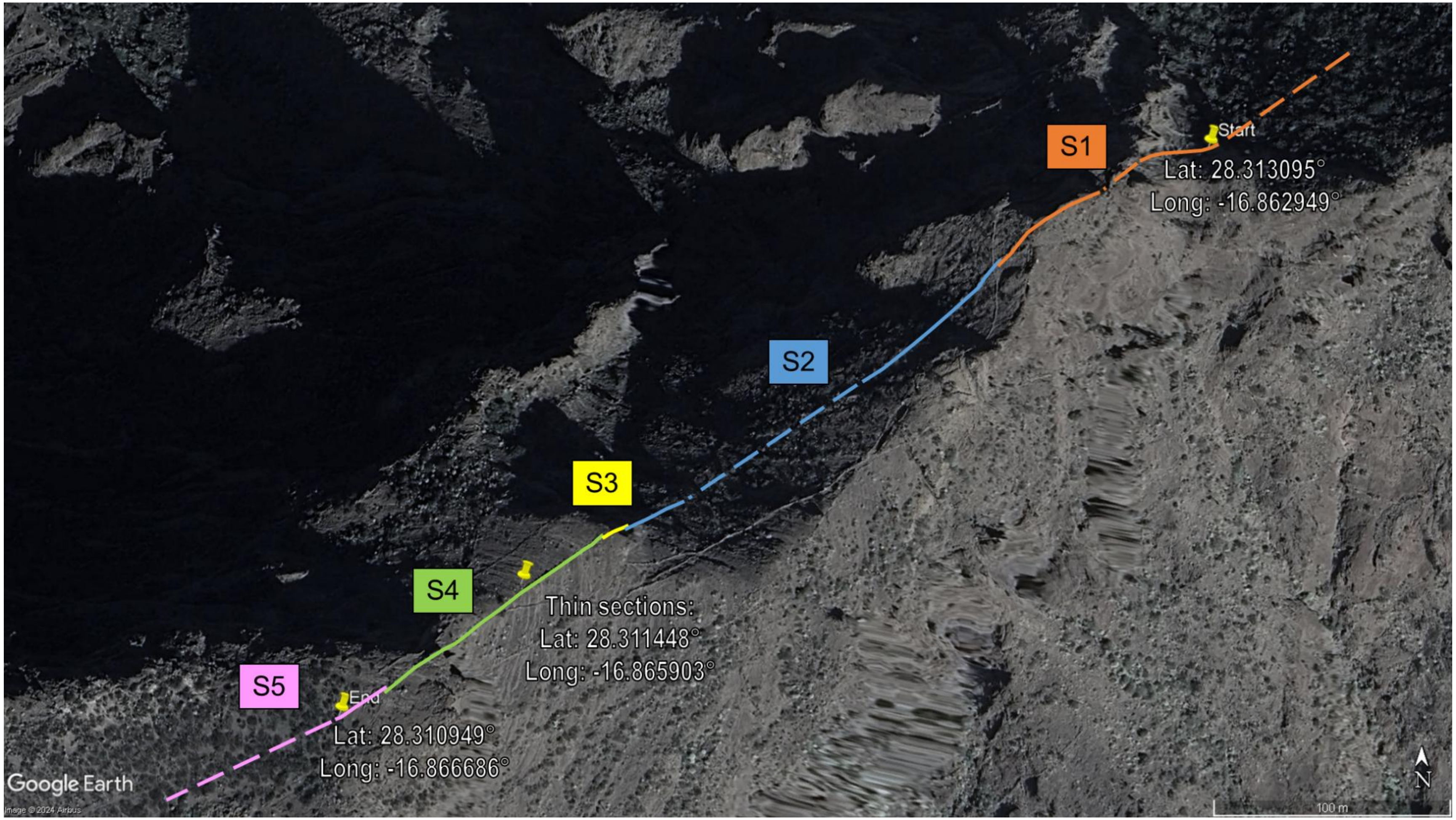

Supplementary Figure 1b. Closer view of study area, showing start and end of studied dyke section, extents of the five identified segments, and location on S4 where thin sections were collected for thin sections. Map data: Google, Airbus.

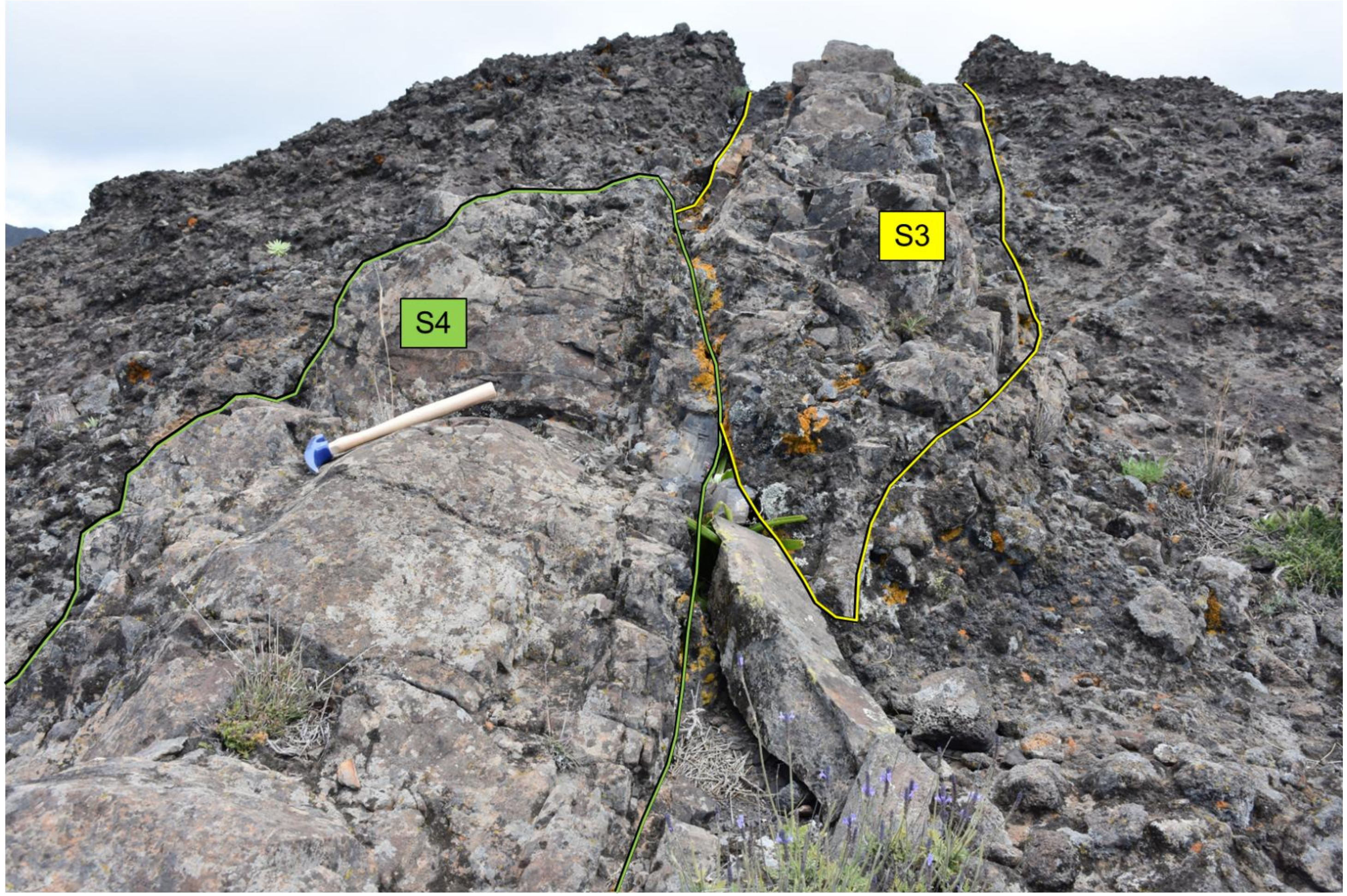

Supplementary Figure 2a. Larger version of Fig. 1d from the main text, showing the relay between S3 and S4, facing NE. The hammer is 30 cm long.

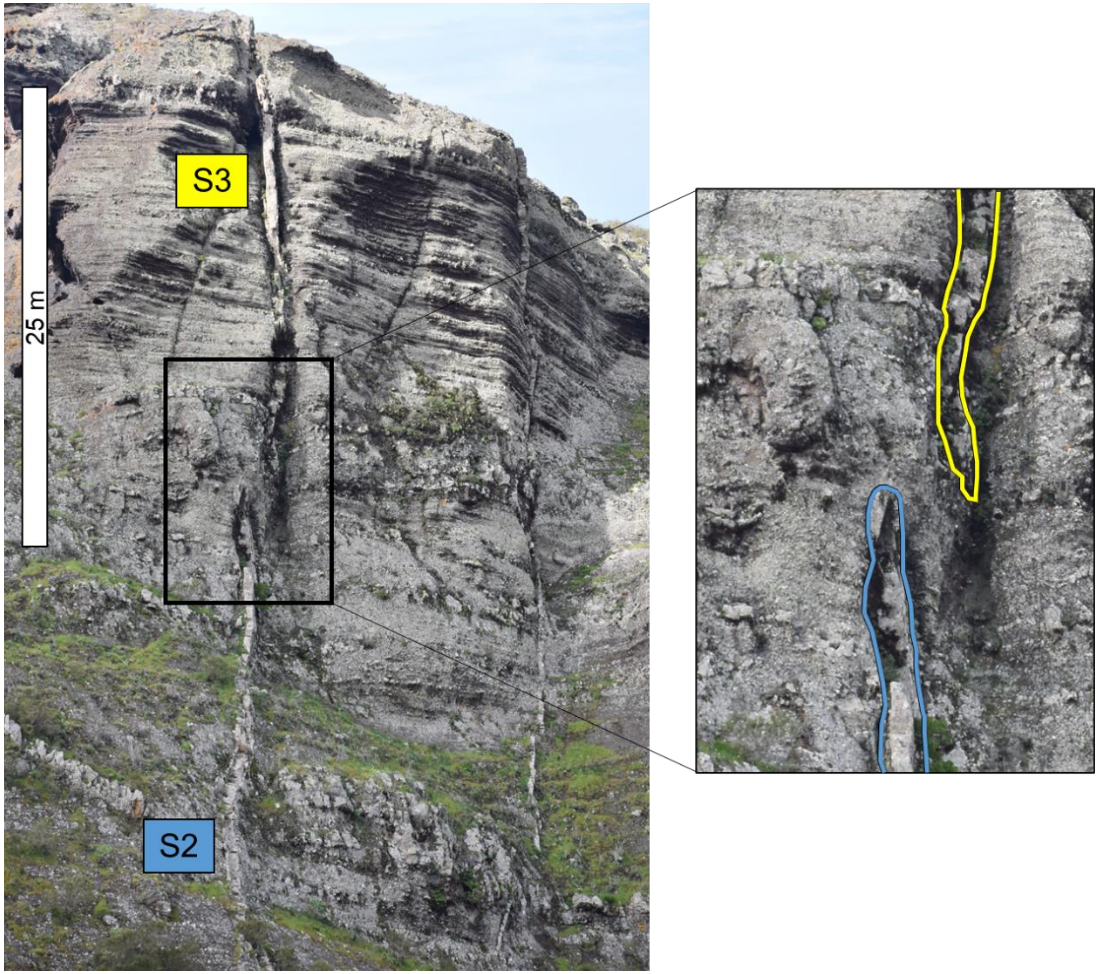

Supplementary Figure 2b. Larger version of Fig. 1e from the main text, showing the relay between S2 and S3, facing SW. The segments are separated by a screen of host rock.

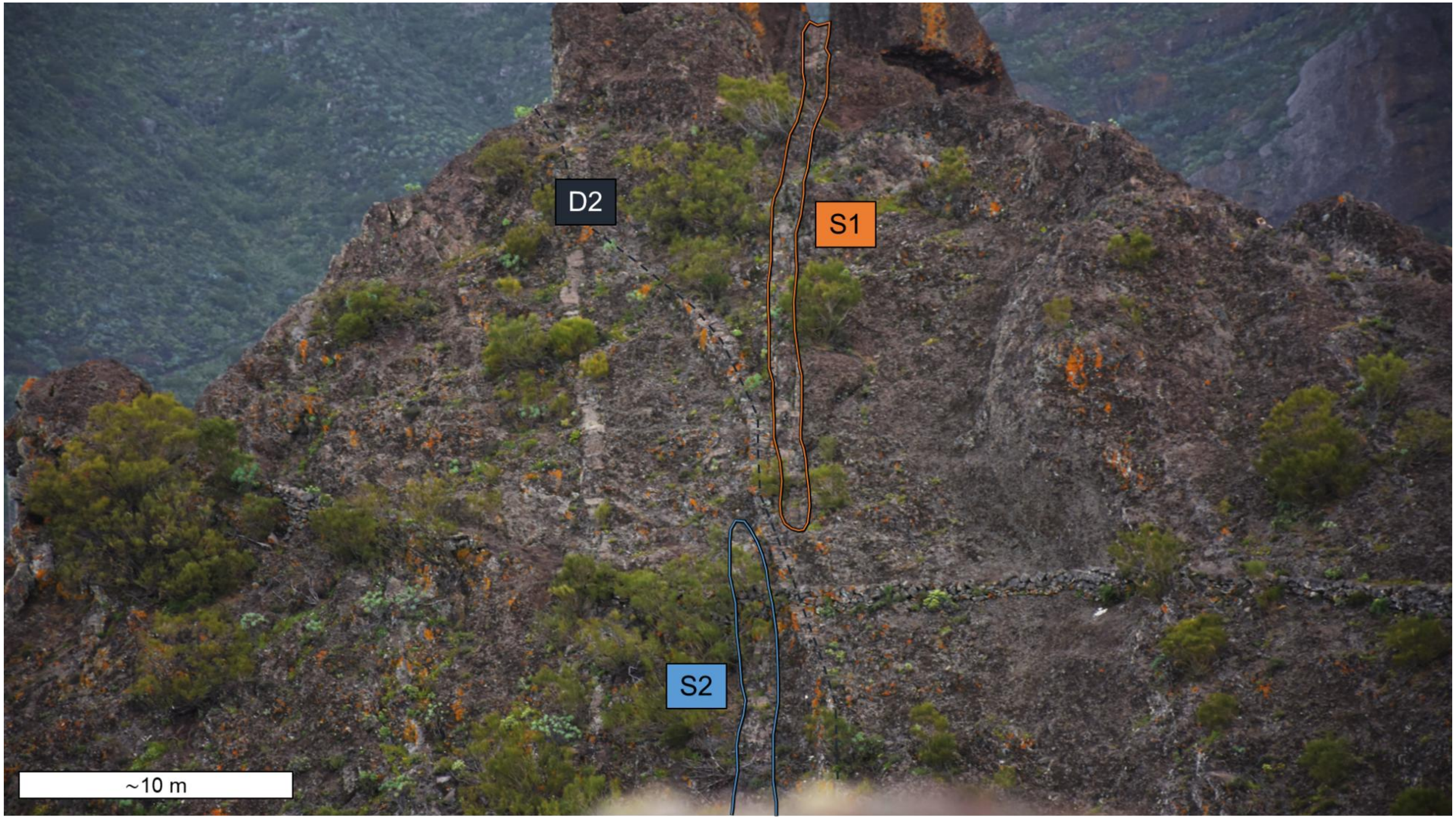

Supplementary Figure 2c. Larger version of Fig. 1f from the main text, showing the relay between S1 and S2, facing NE from the top of the cliff shown in Supplementary Figure 2c (Fig. 1e in main text). Segments S1 and S2 are separated by another dyke, D2.

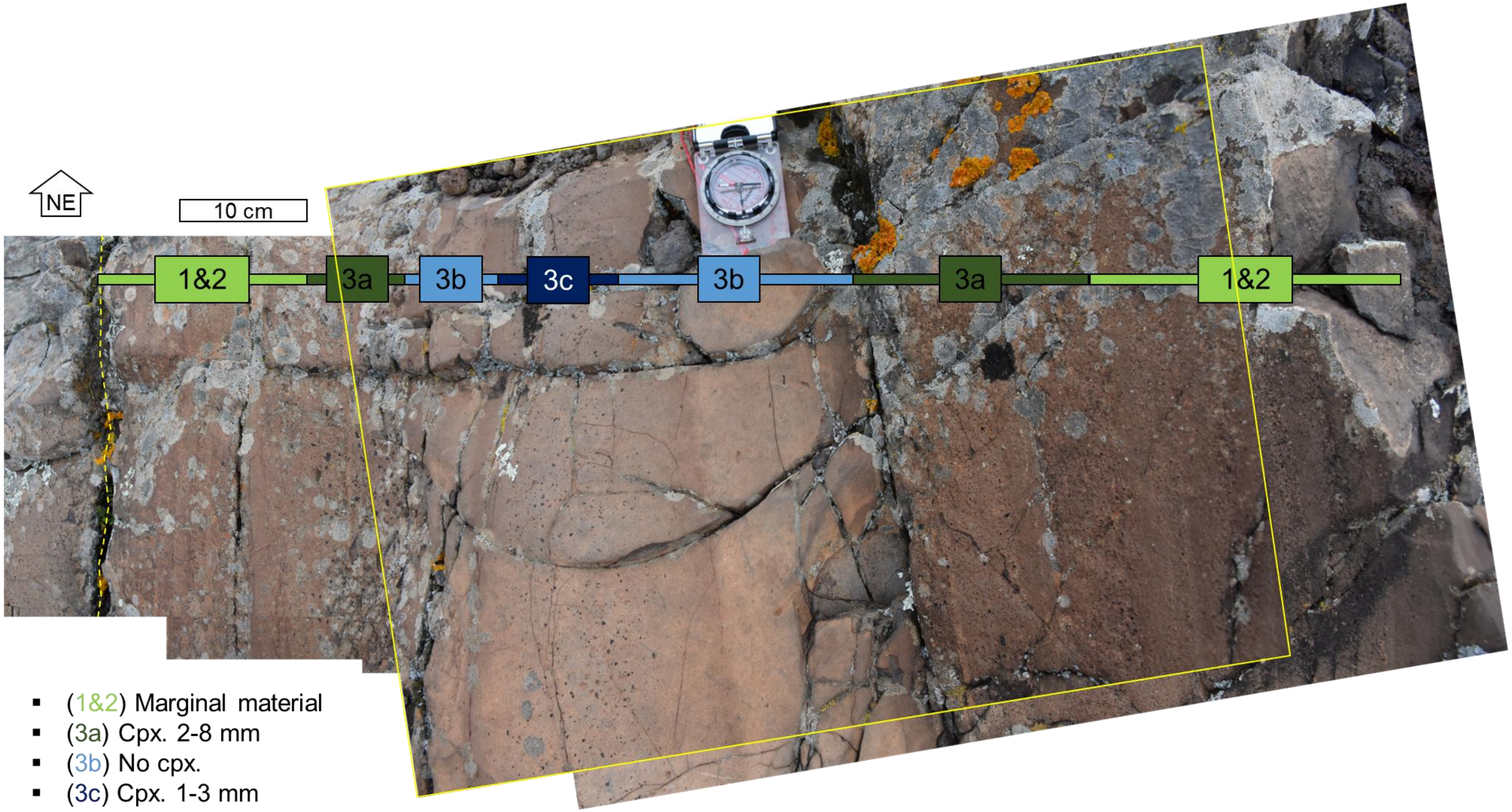

Supplementary Figure 3a. Larger version of Fig. 3e from the main text, showing the photo montage of textural layers and sub-layers within S4. A dashed yellow line shows the margin on the north side. The image with the yellow outline is shown at higher resolution in Supplementary Figure 3b. In the layer descriptions, “cpx” refers to clinopyroxene phenocrysts.

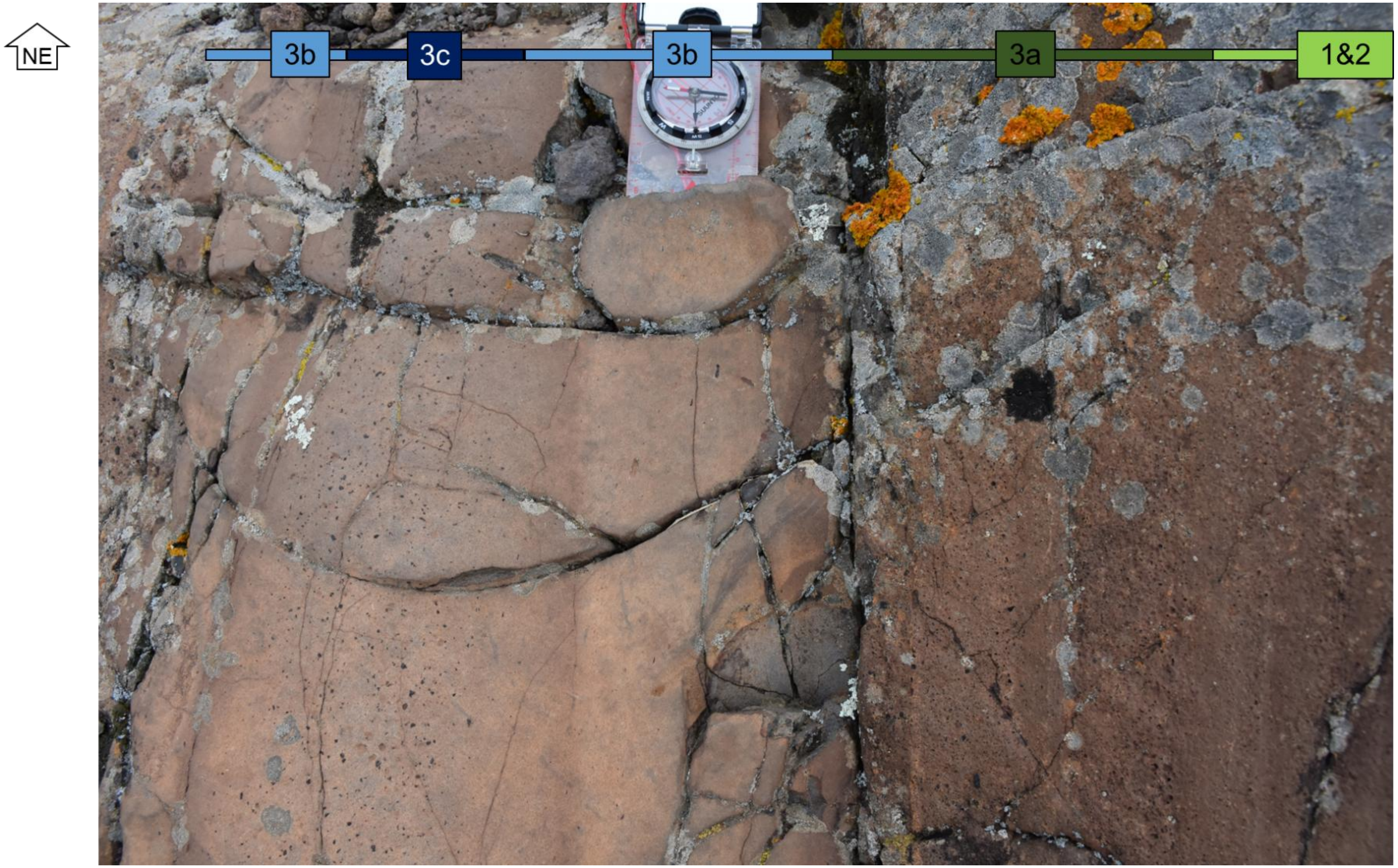

Supplementary Figure 3b. Close-up of the central sub-layers in S4, from the photo montage presented as Fig. 3e in the main text. For sub-layer descriptions, see caption for Supplementary Figure 3a.

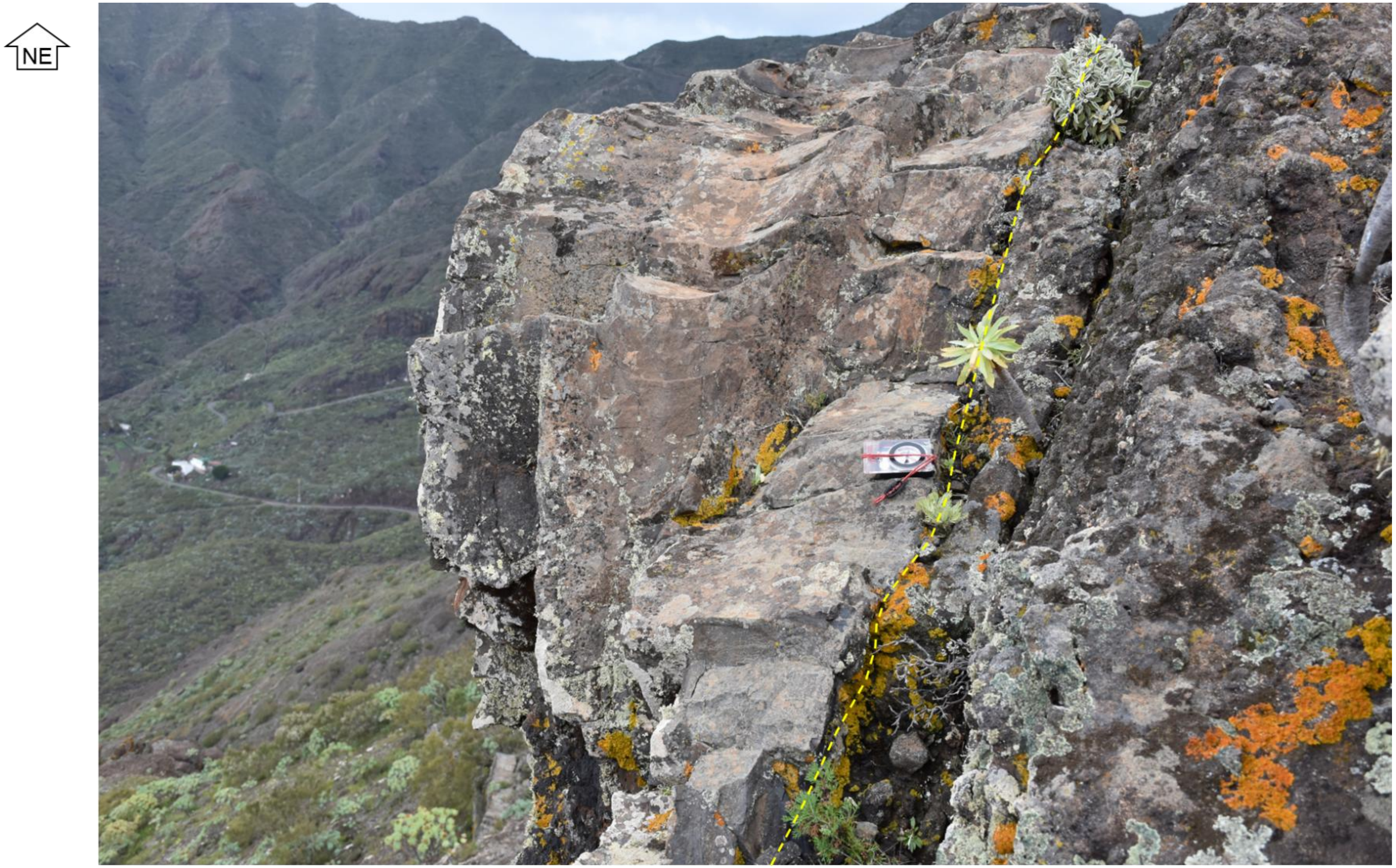

Supplementary Figure 3c. Larger version of Fig. 3d from the main text, showing textural layers within S4. Compass is approximately 10 cm long. Yellow dashed line shows dyke margin. Facing NE.

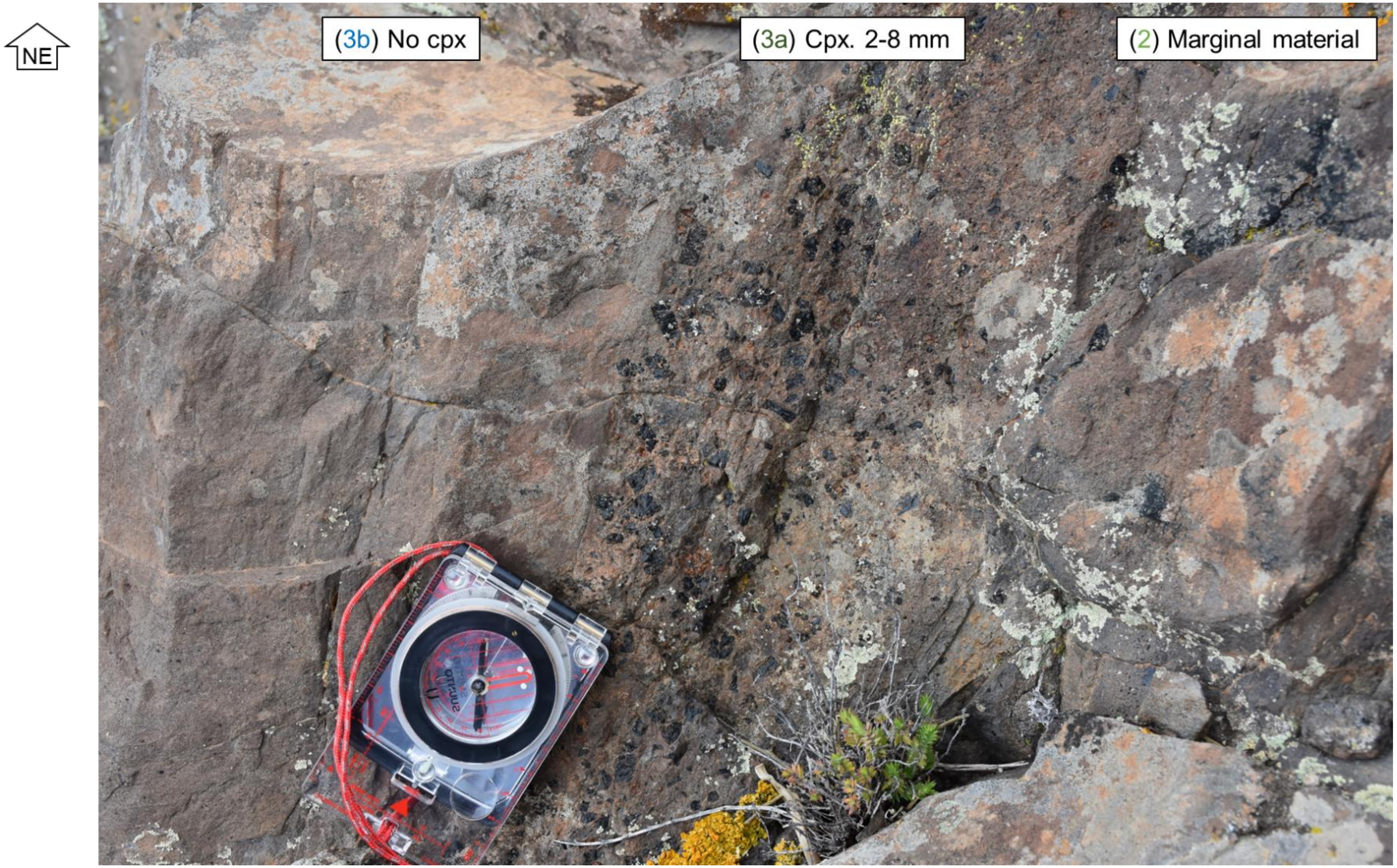

Supplementary Figure 3d. Larger version of Fig. 3d inset from the main text, showing textural layers within S4. Compass is approximately 10 cm long. Facing NE. Dyke centre is to the left, and dyke margin is to the right. “Cpx” refers to clinopyroxene phenocrysts.

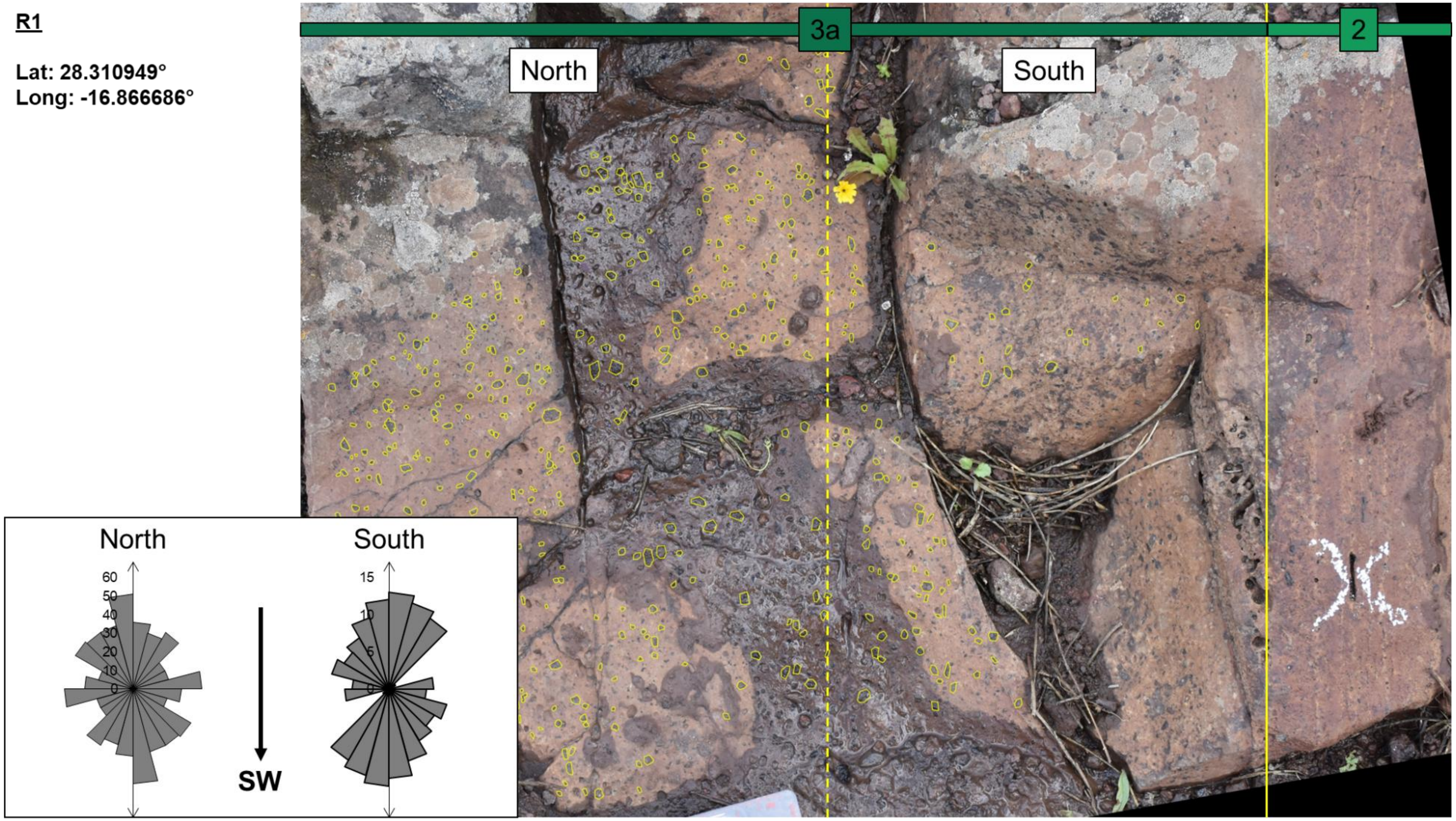

Supplementary Figure 4a. Image containing phenocryst outlines at site R1. Orientations of phenocryst long axes are displayed on rose diagrams, shown in Fig. 4 in main text, split into north and south halves of the dyke. Crystal orientations imply a SW flow component.

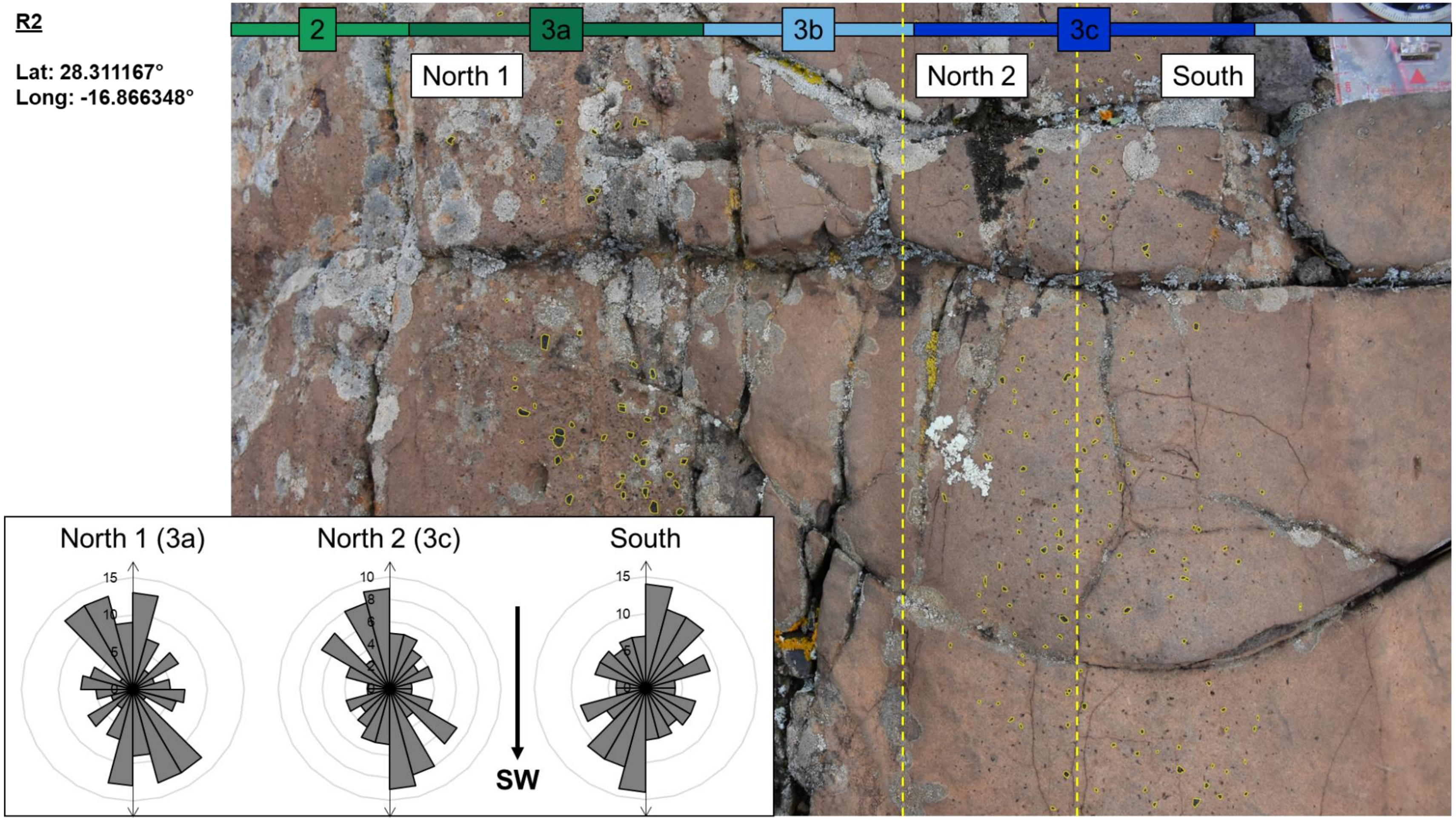

Supplementary Figure 4b. Image containing phenocryst outlines at site R2, used to construct rose diagrams presented in Fig. 4 in main text. Here, orientations are split further into two northern groups, from sublayers 3a and 3c.

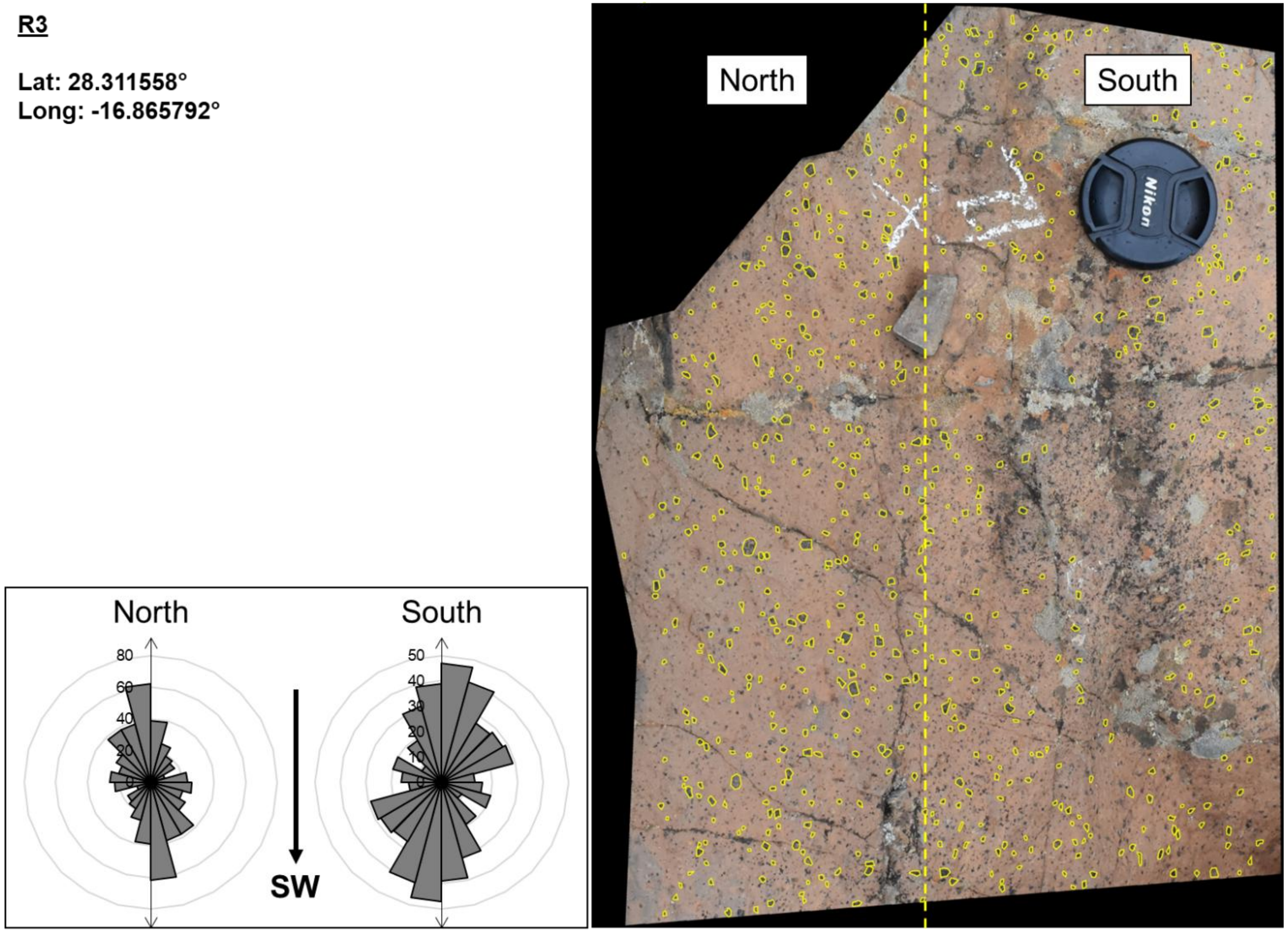

Supplementary Figure 4c. Image containing phenocryst outlines at site R3, used to construct rose diagrams presented in Fig. 4 in main text.

R4

Lat: 28.311678°
Long: -16.865631°

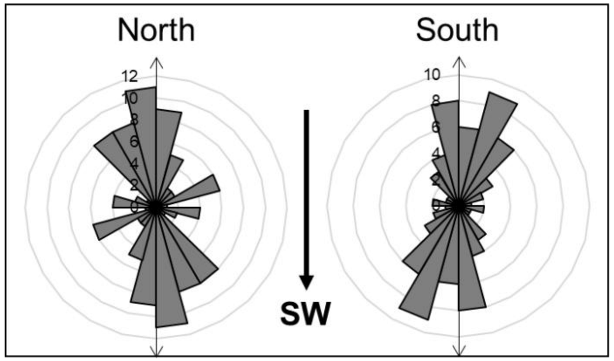

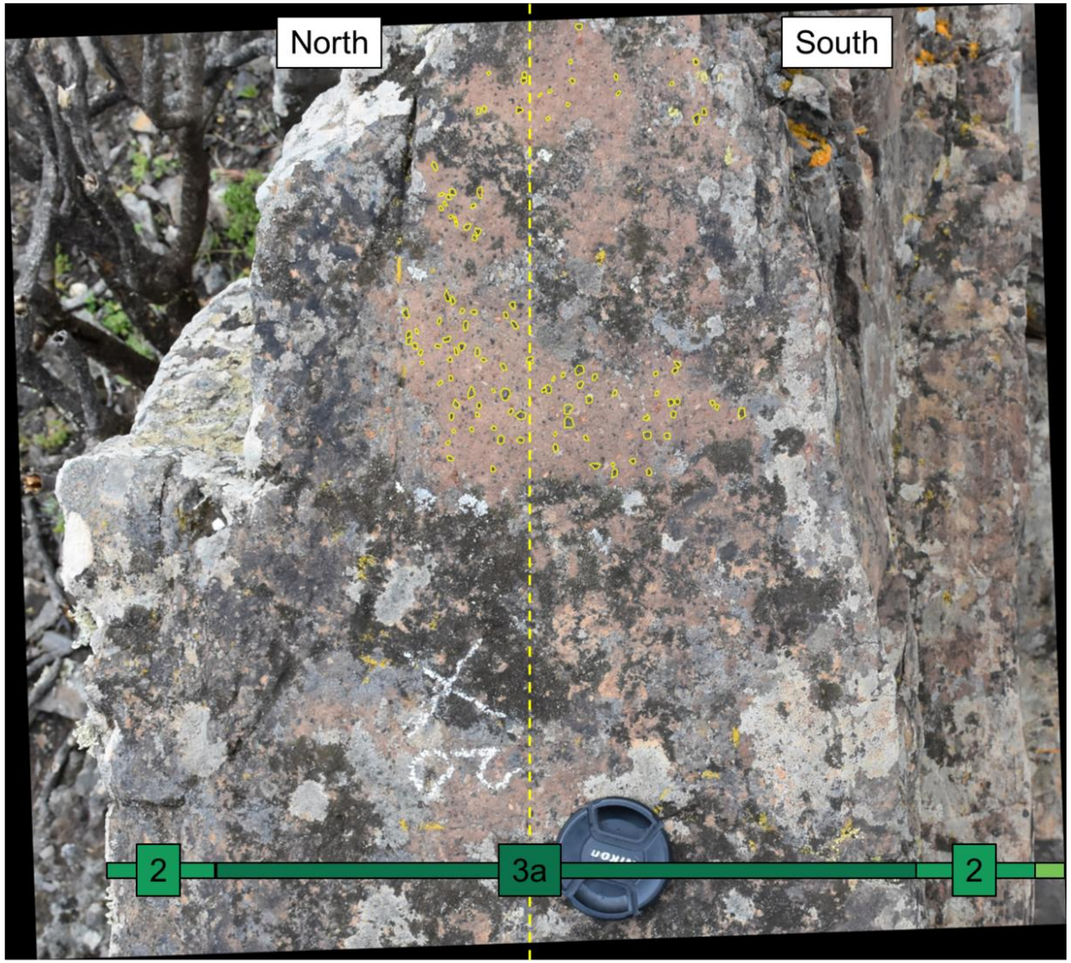

Supplementary Figure 4d. Image containing phenocryst outlines at site R4, used to construct rose diagrams presented in Fig. 4 in main text.

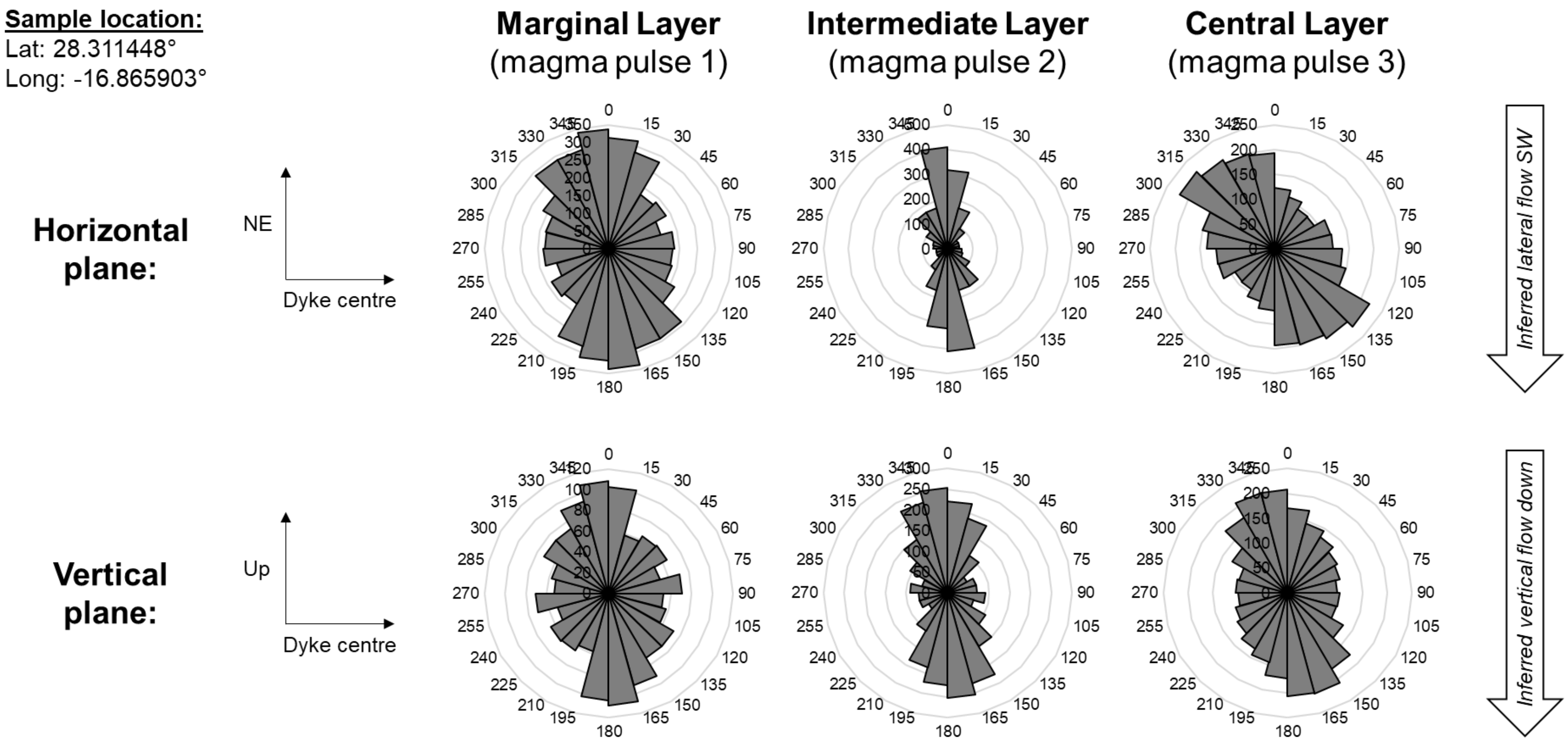

Supplementary Figure 5. Larger versions of rose diagrams shown in Fig. 4c in main text. Orientations of microlites in the three layers of S4, identified in thin section images by thresholding based on brightness. Top row shows horizontal plane, with NE at 0°, and bottom row shows vertical plane, with up at 0°. Both rows have the dyke centre at 90°.

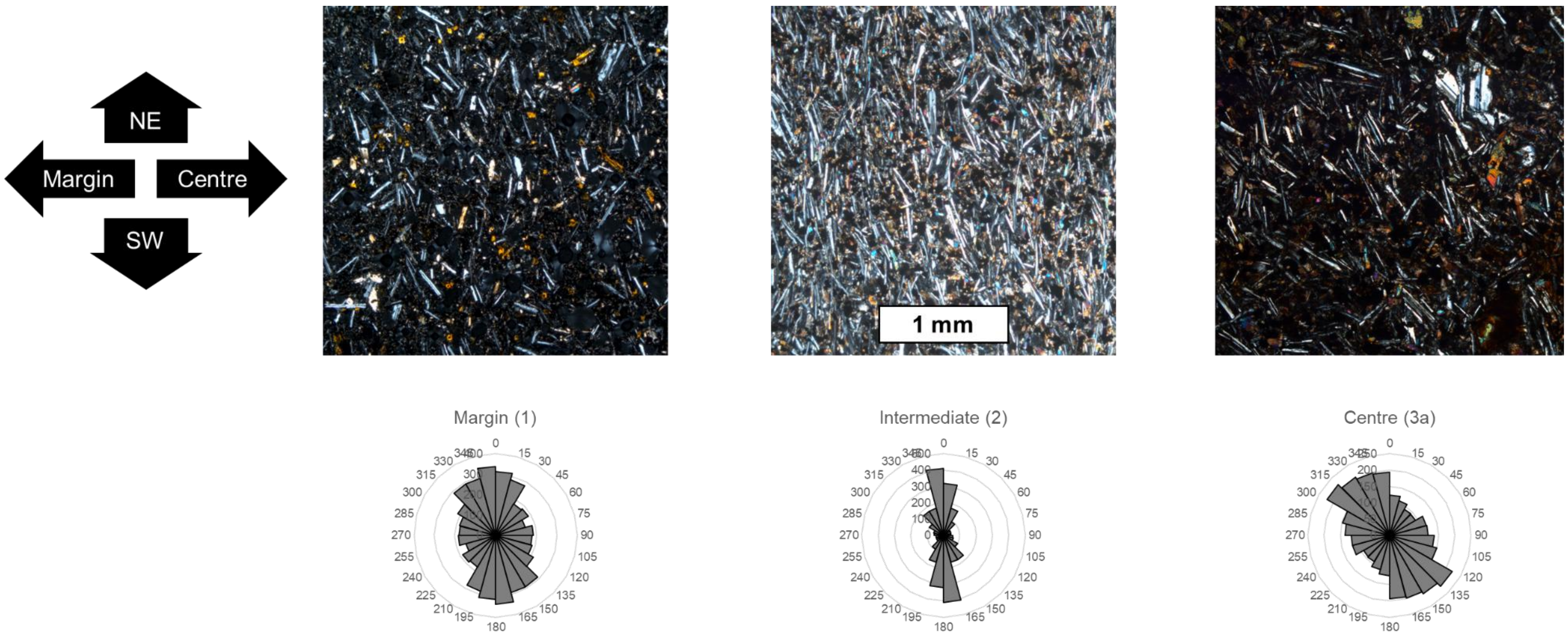

Supplementary Figure 6a. Copies of the rose diagrams shown in Fig. 4c in the main text, with cropped regions of thin sections from which the microlite orientation data were collected. Thin sections are from the horizontal plane, in cross-polarised light. All images have the same scale. All three layers contain imbrication textures implying the same direction of horizontal flow. Larger versions of the photomicrographs are presented in Supplementary Figures 7-9.

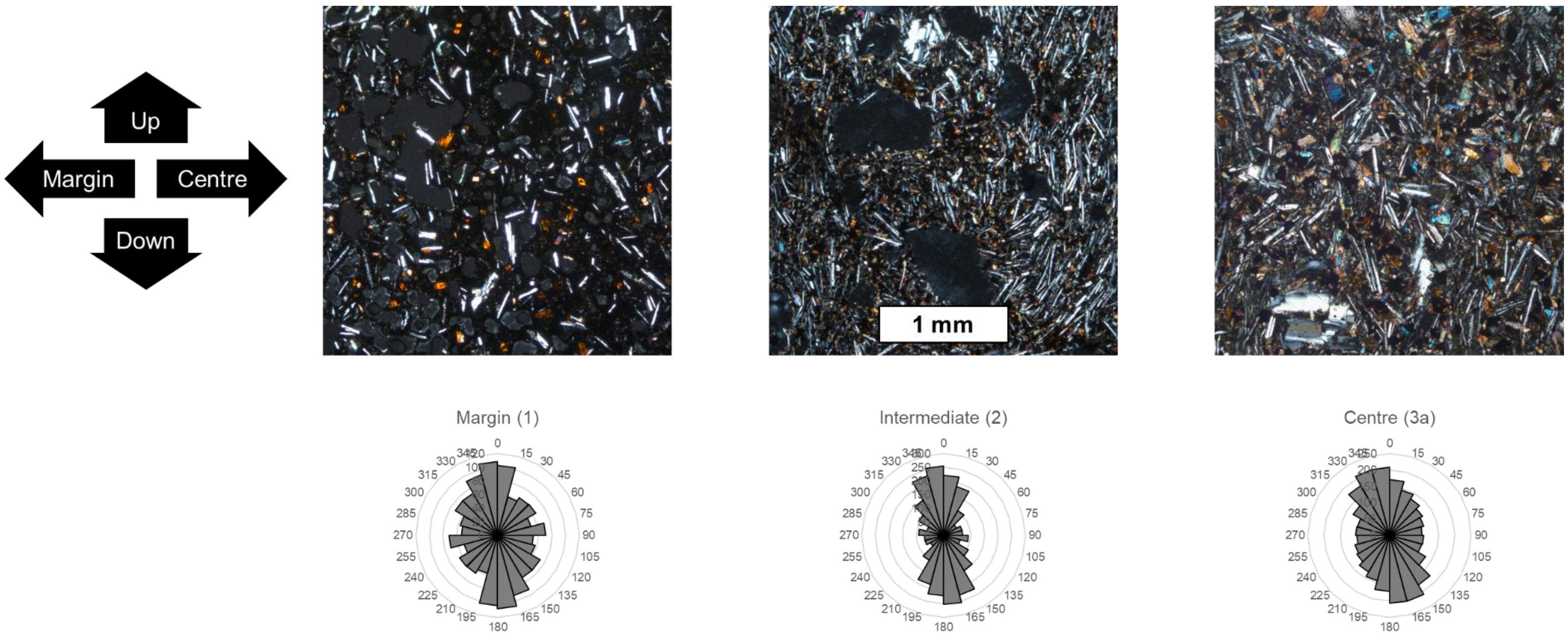

Supplementary Figure 6b. Copies of the rose diagrams shown in Fig. 4c in main text, with cropped regions of the thin sections from which the microlite orientation data were collected. Thin sections are from the vertical plane, in cross-polarised light. All images have the same scale. All three layers contain imbrication textures implying the same direction of vertical flow. Larger versions of the photomicrographs are presented in Supplementary Figures 7-9.

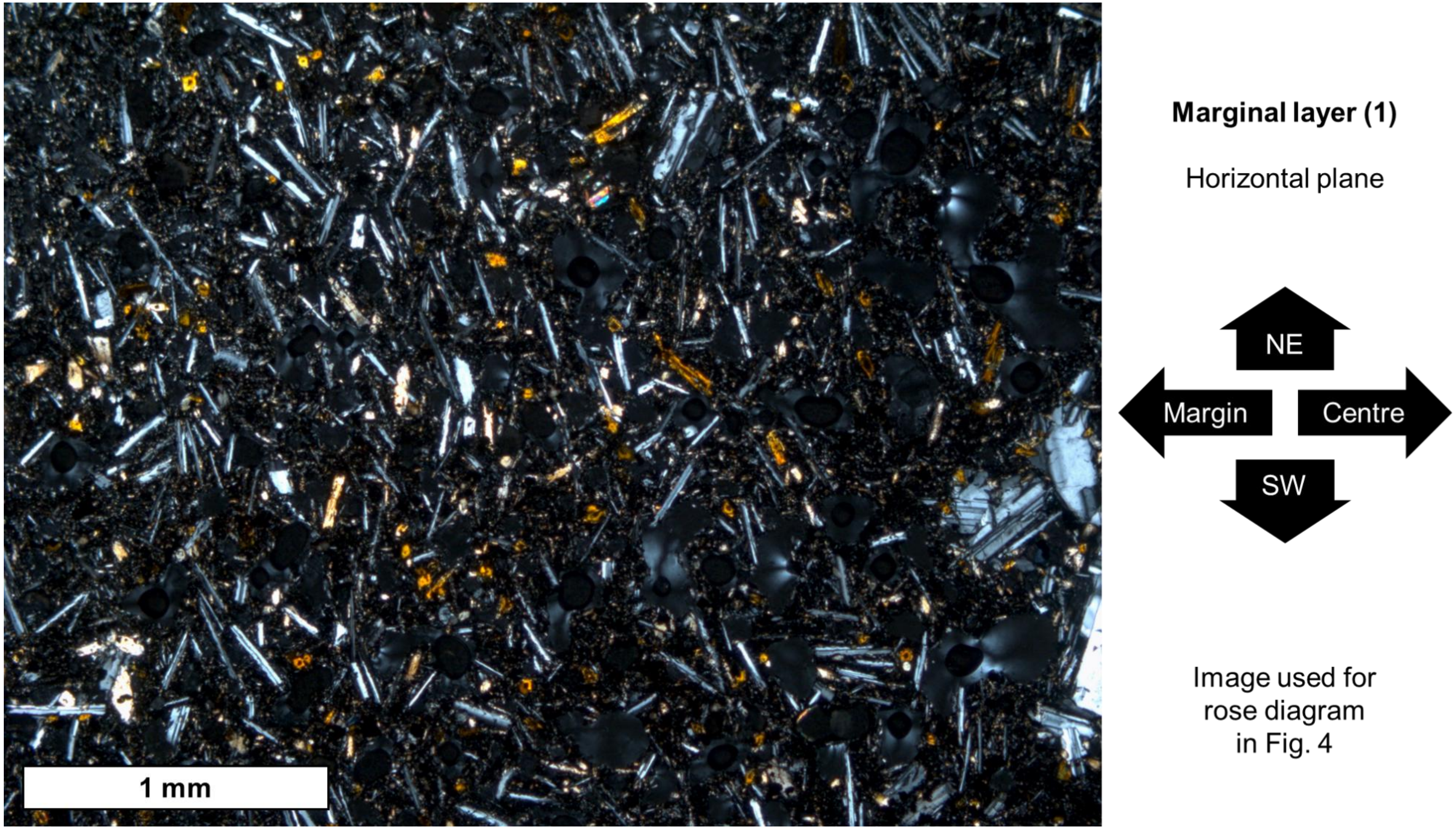

Supplementary Figure 7a. Photomicrograph from marginal layer of segment S4, horizontal plane, cross-polarised light.

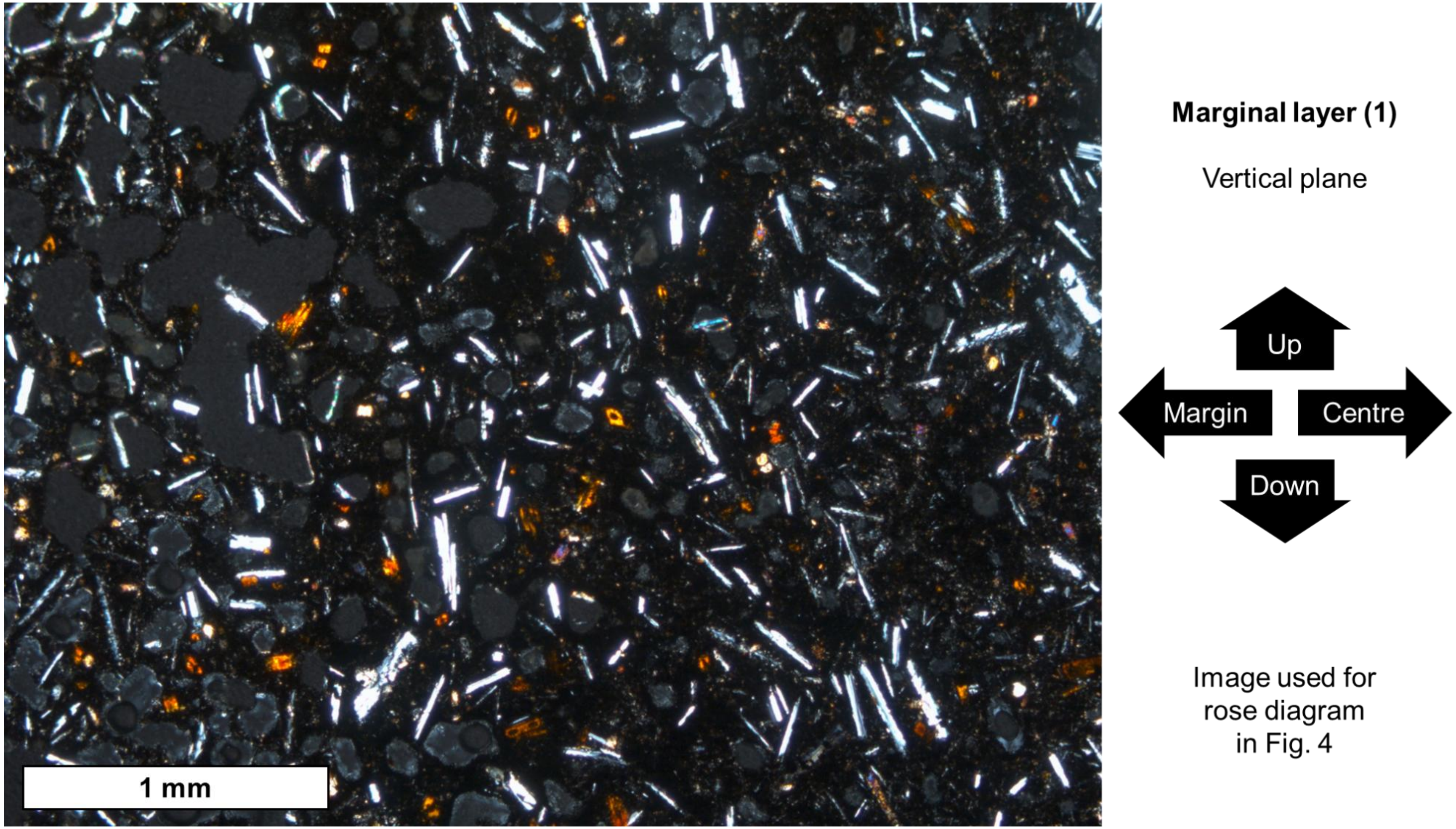

Supplementary Figure 7b. Photomicrograph from marginal layer of segment S4, vertical plane, cross-polarised light.

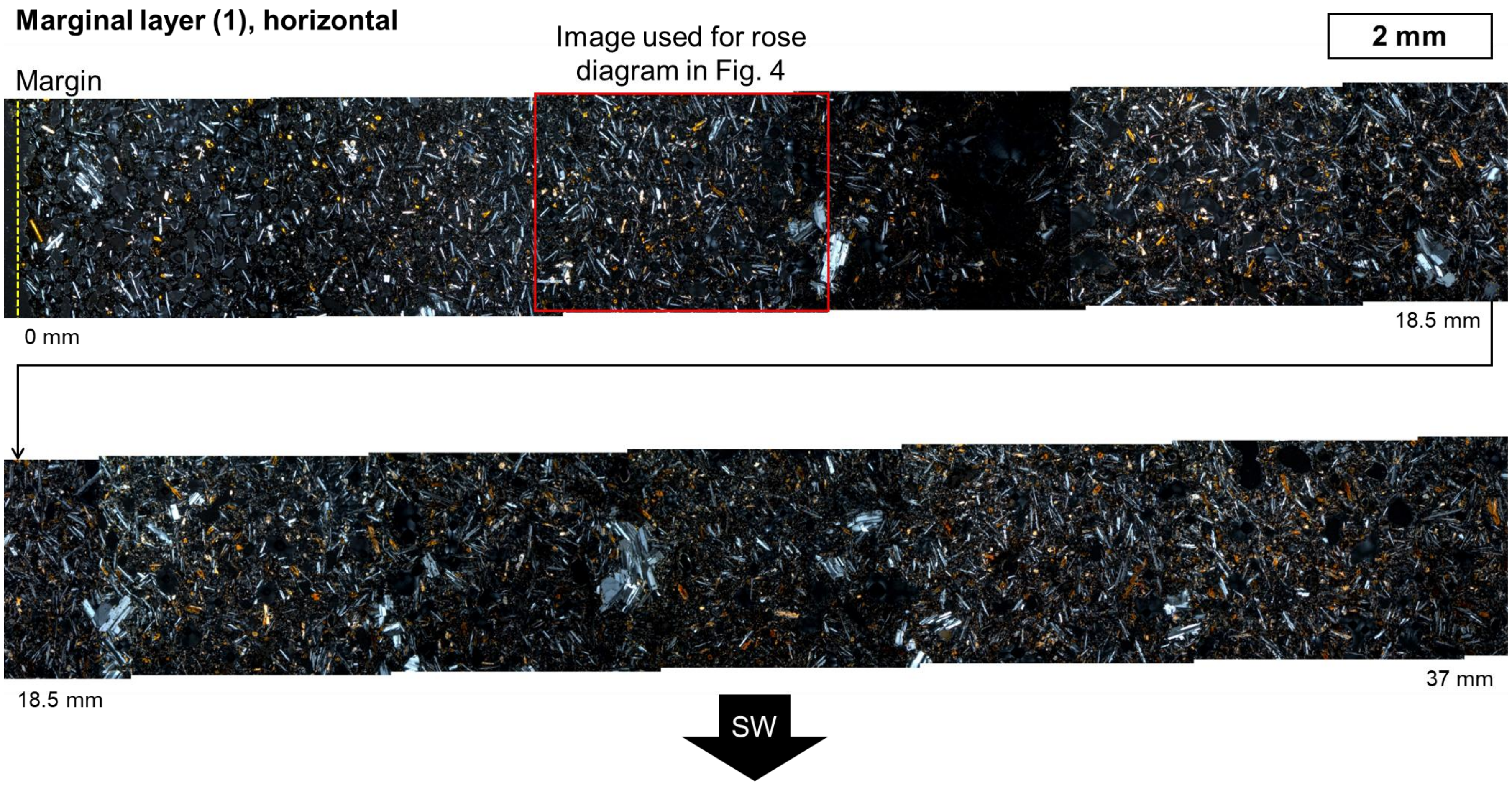

Supplementary Figure 7c. Photomicrograph swaths from marginal layer of segment S4, horizontal plane, cross-polarised light, moving inwards from the margin, which is shown with a dashed yellow line. The image in the red box was used for the rose diagrams in Figure 4, and presented as Supplementary Figure 7a.

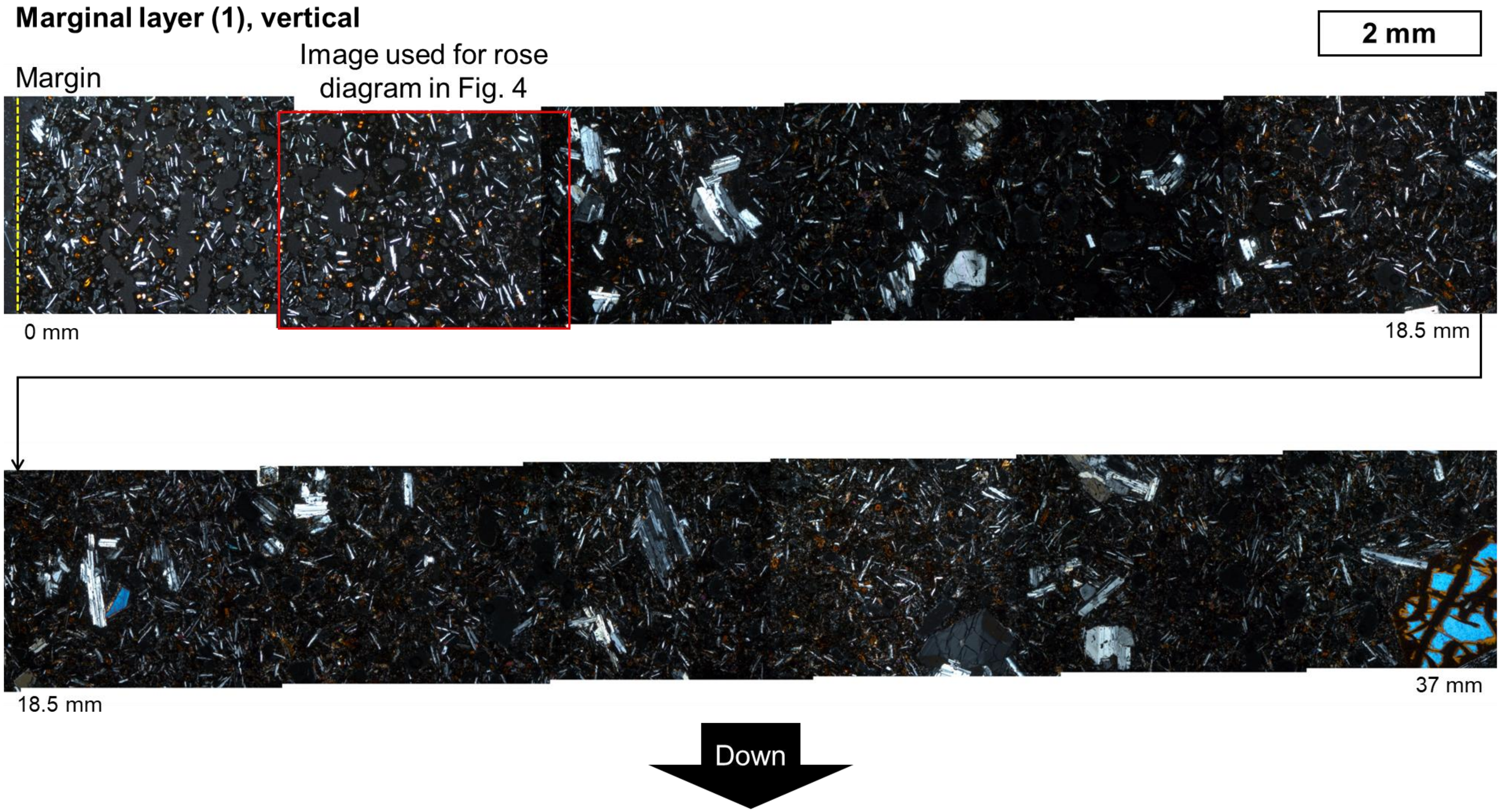

Supplementary Figure 7d. Photomicrograph swaths from marginal layer of segment S4, vertical plane, cross-polarised light, moving inwards from the margin, which is shown with a dashed yellow line. The image in the red box was used for the rose diagrams in Figure 4, and presented as Supplementary Figure 7b.

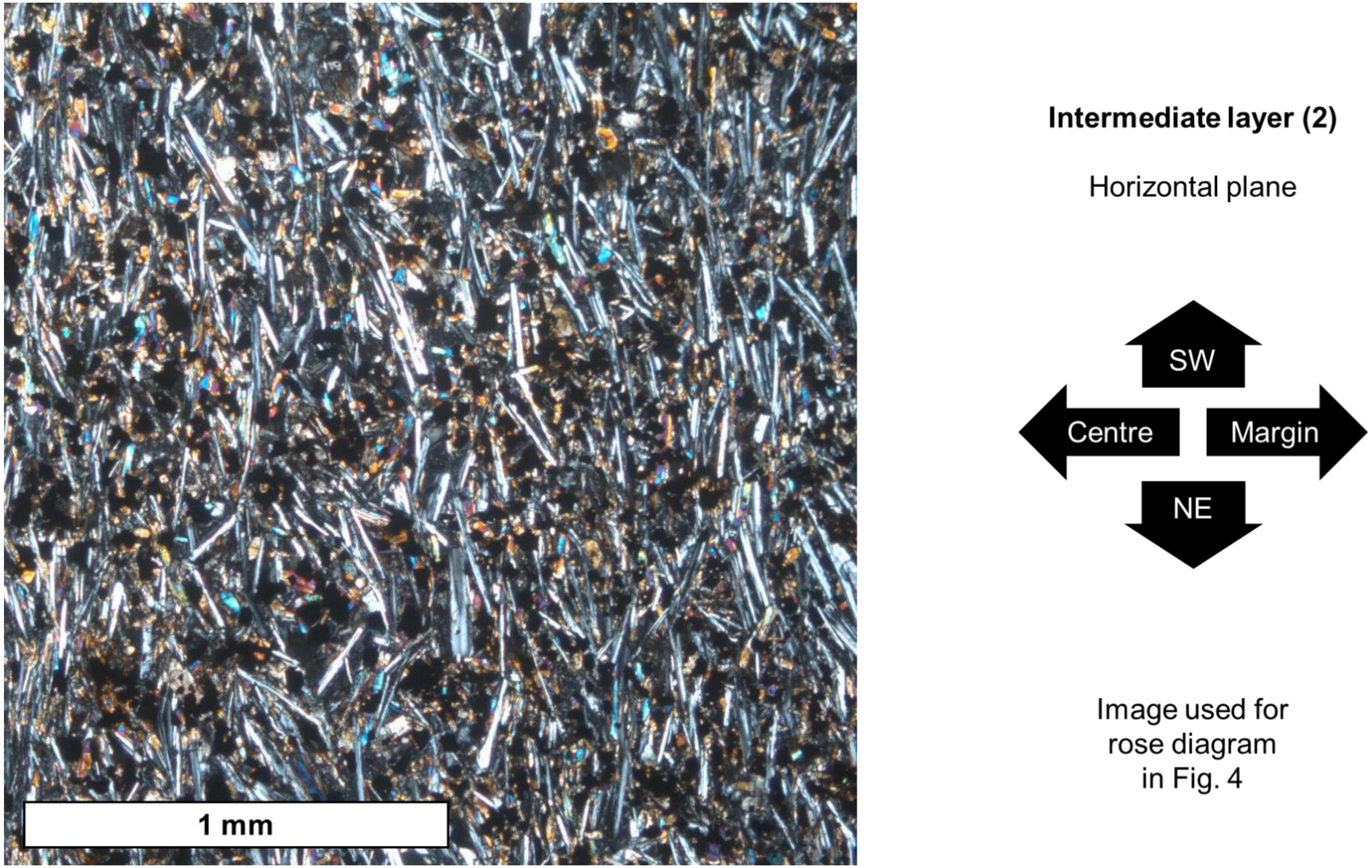

Supplementary Figure 8a. Photomicrograph from the intermediate layer of segment S4, horizontal plane, cross-polarised light.

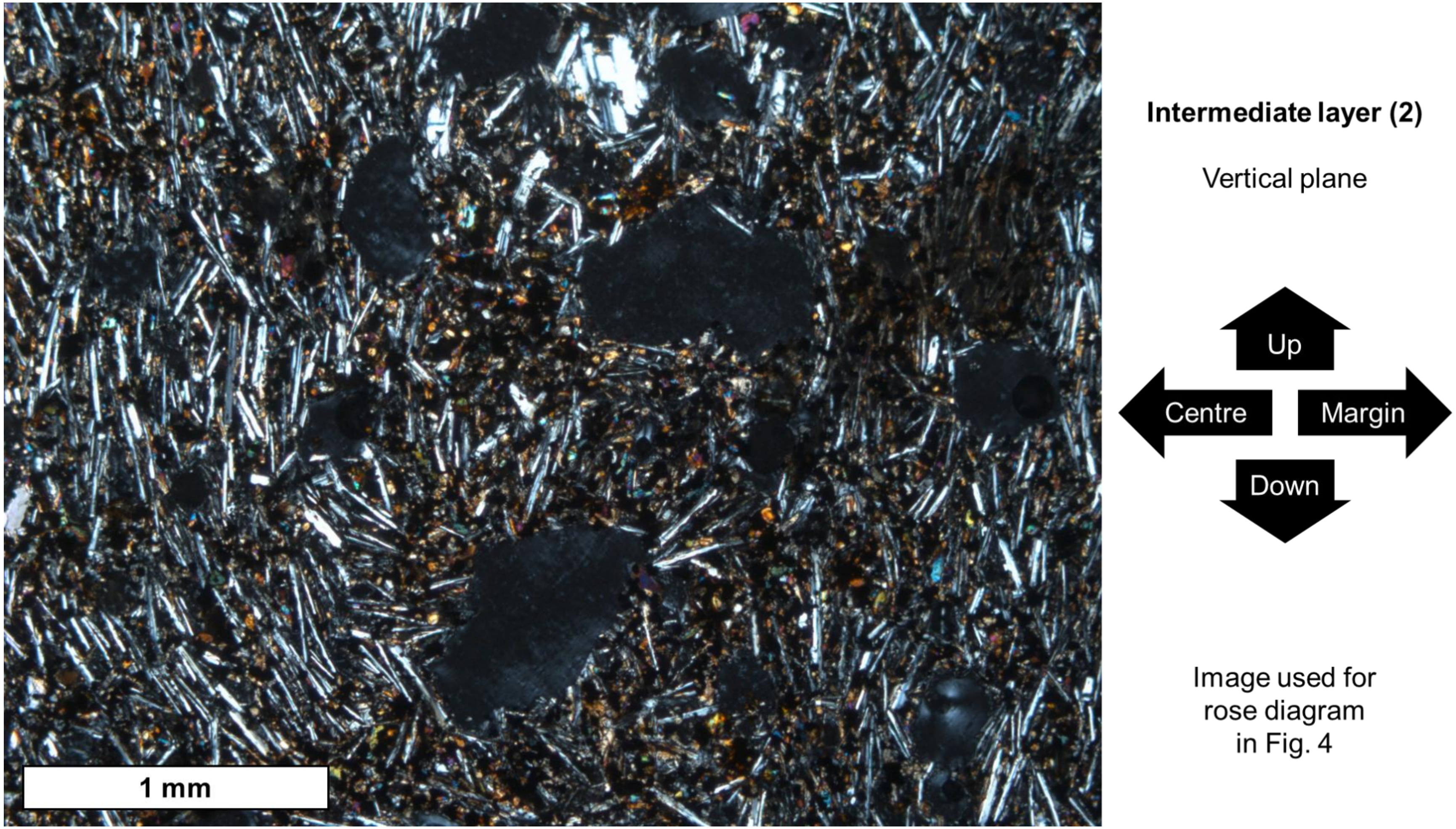

Supplementary Figure 8b. Photomicrograph from the intermediate layer of segment S4, vertical plane, cross-polarised light.

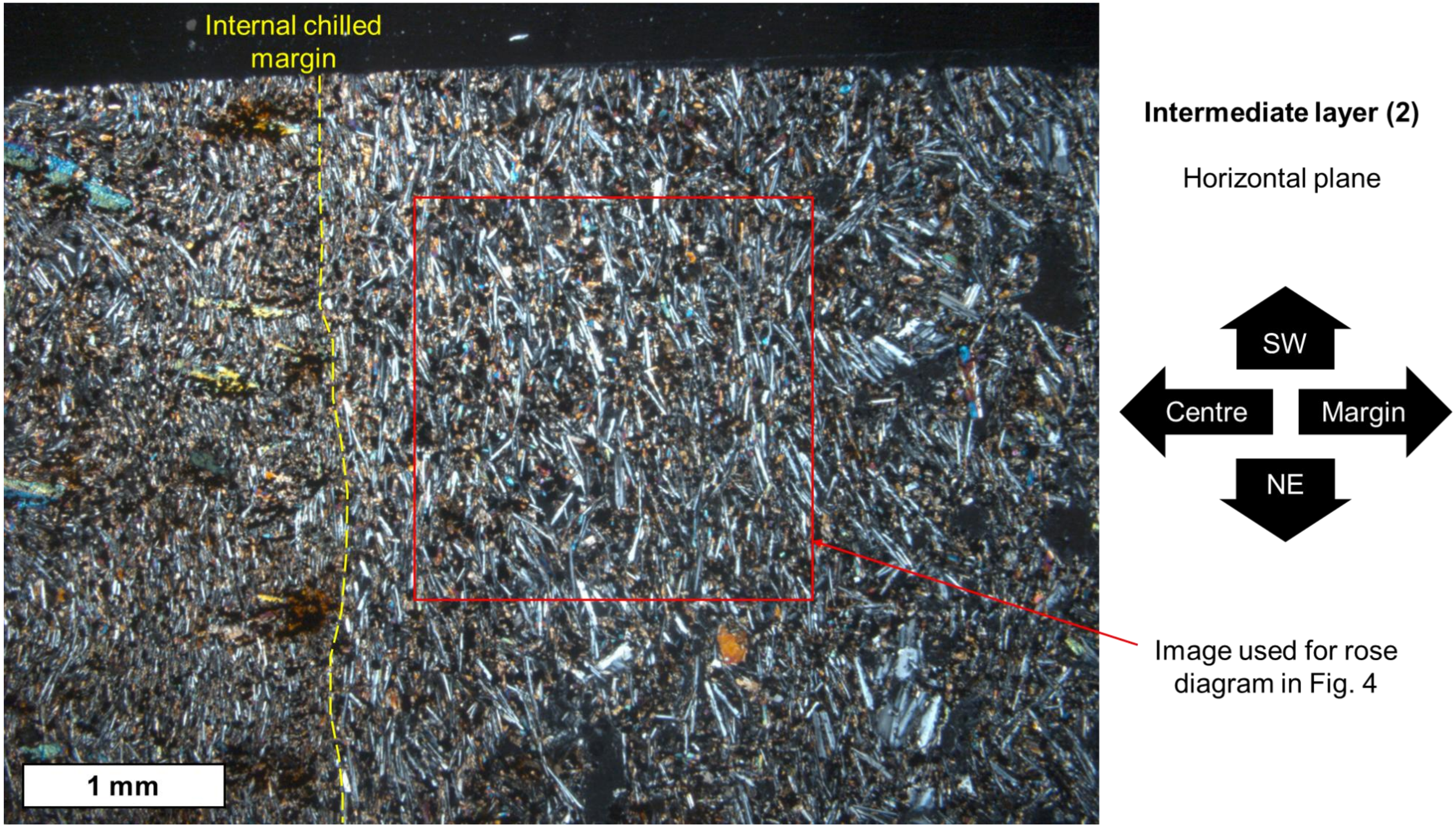

Supplementary Figure 8c. Photomicrograph from the intermediate layer of segment S4, horizontal plane, cross-polarised light. The internal chilled margin at the boundary with the central layer is shown with a yellow dashed line. This is presented at higher magnification in Supplementary Figure 10.

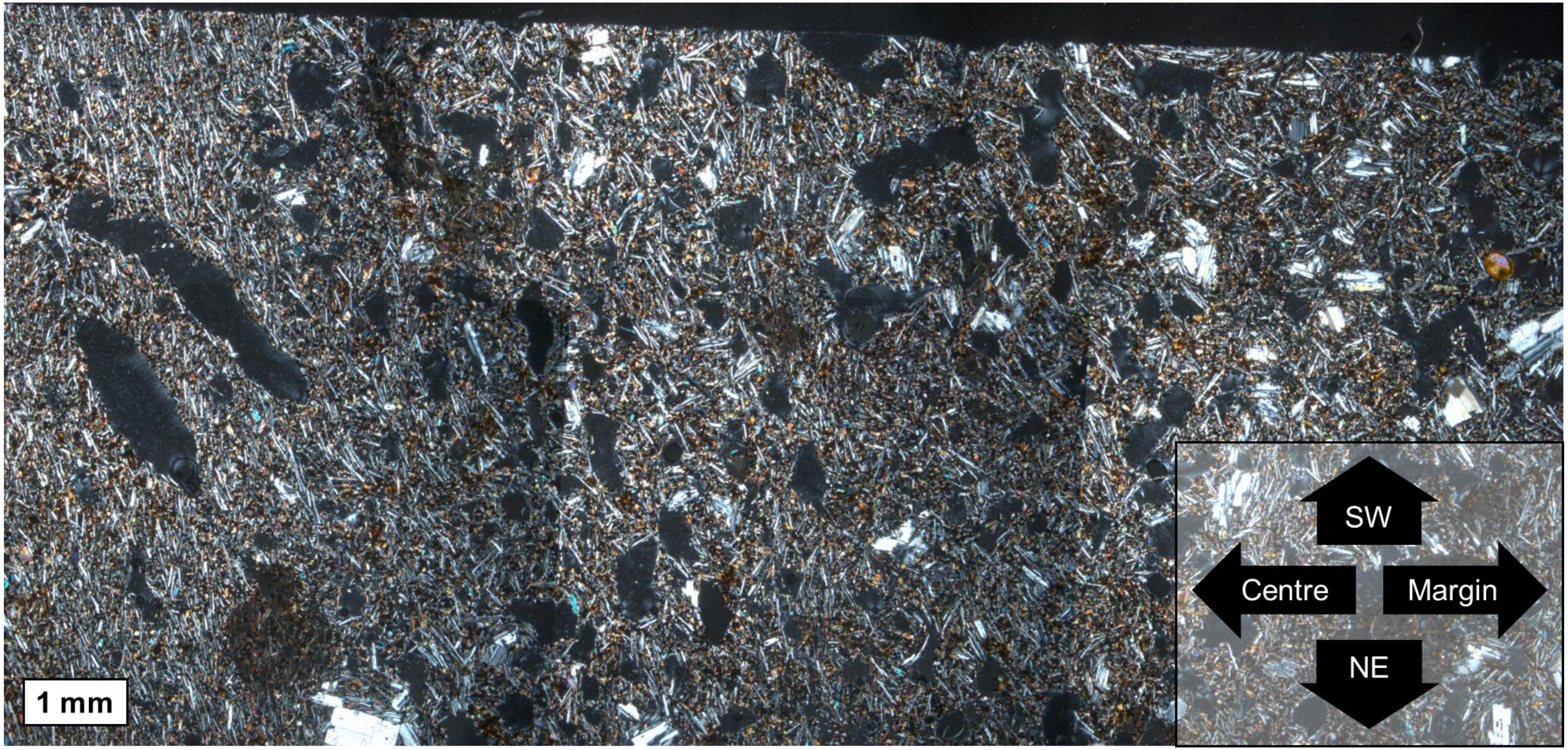

Supplementary Figure 8d. Photomicrograph montage from the intermediate layer of segment S4, horizontal plane, cross-polarised light, showing elongated vesicles stretched out to indicate a SW horizontal flow component.

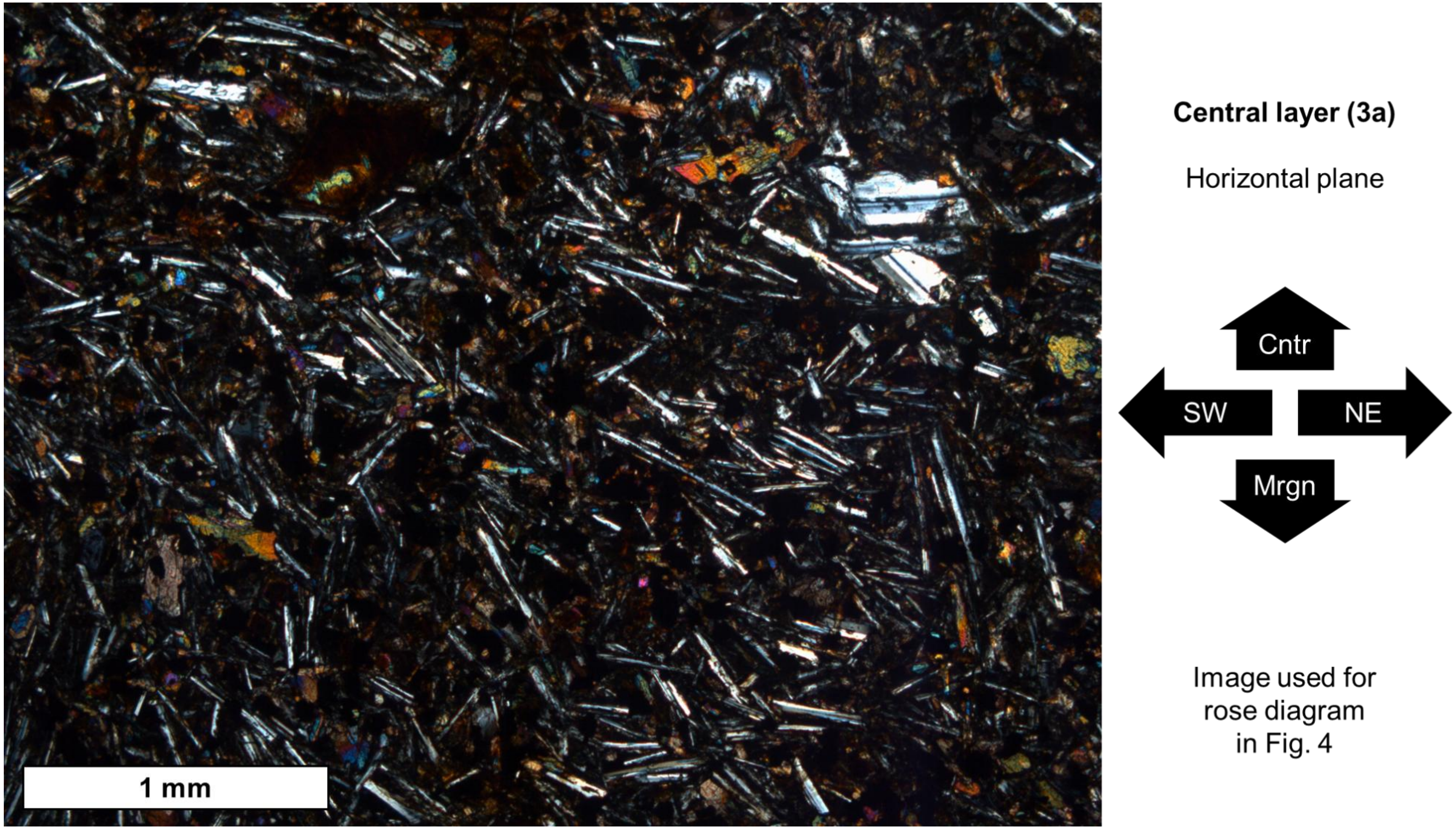

Supplementary Figure 9a. Photomicrograph from the central layer of segment S4, horizontal plane, cross-polarised light.

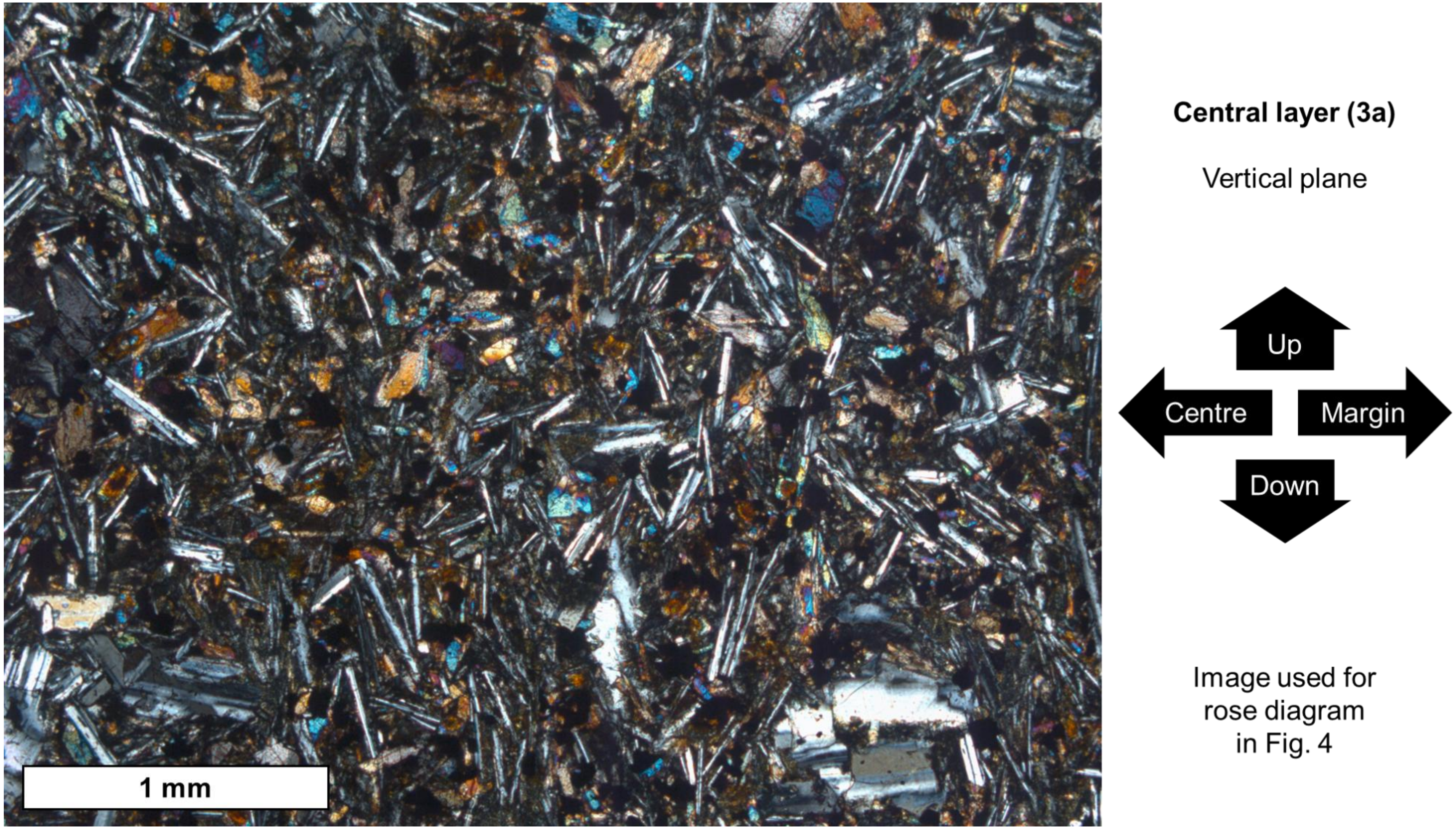

Supplementary Figure 9b. Photomicrograph from the central layer of segment S4, vertical plane, cross-polarised light.

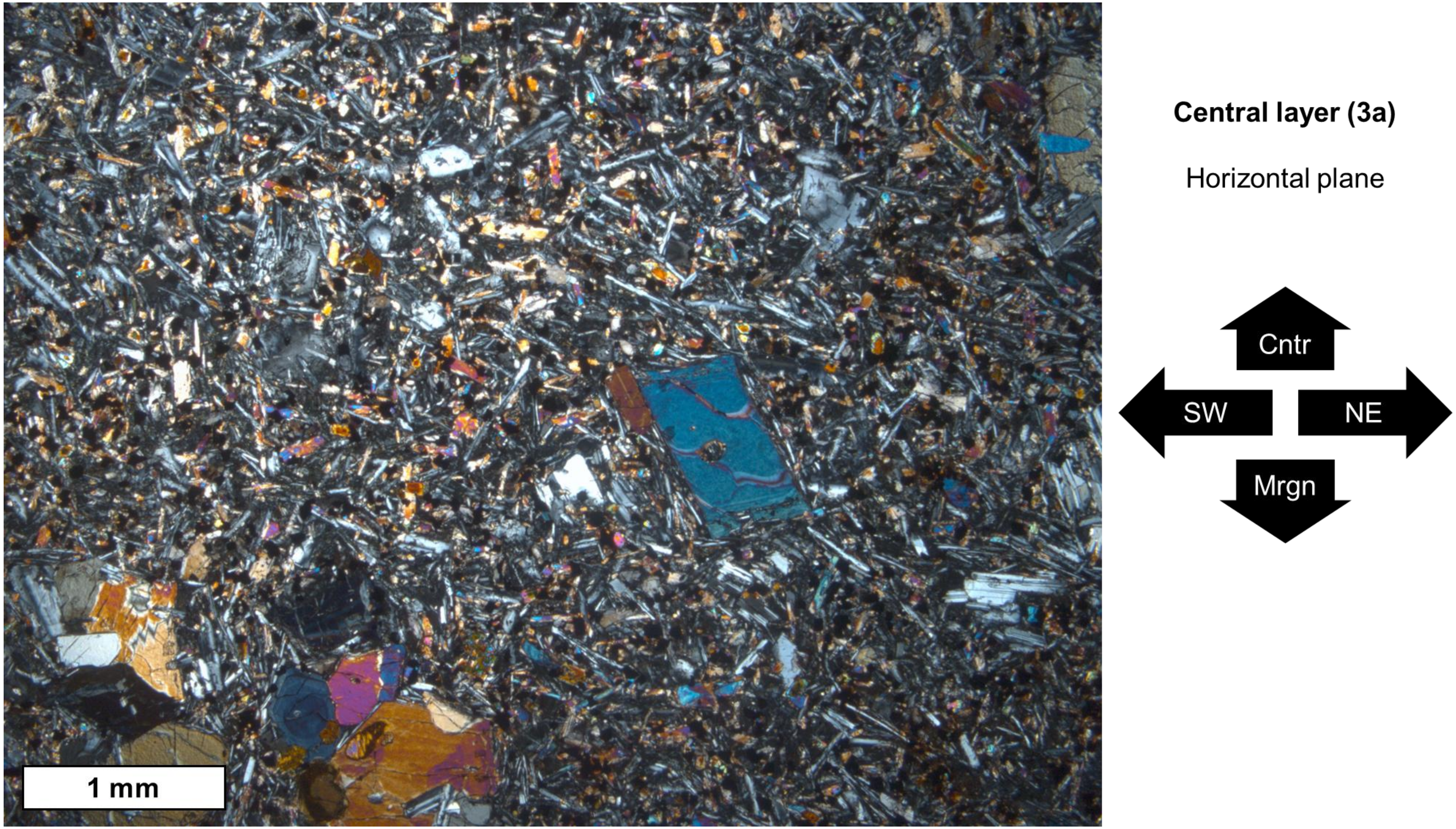

Supplementary Figure 9c. Photomicrograph from the central layer of segment S4, horizontal plane, cross-polarised light, at lower magnification for a larger field of view.

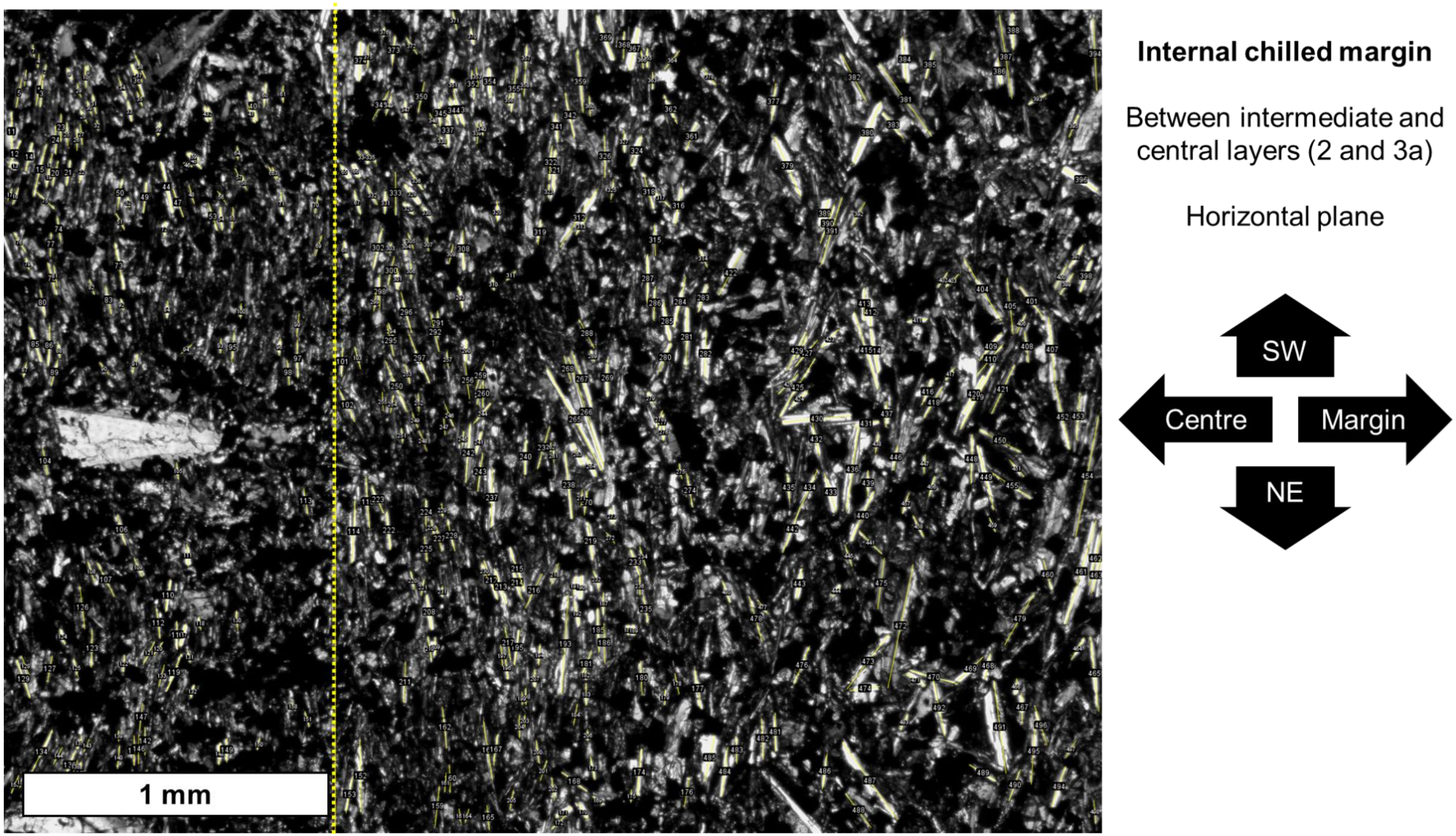

Supplementary Figure 10a. Greyscale photomicrograph from the chilled margin between intermediate and central layers, originally in cross-polarised light. Internal chilled margin is marked with a dashed yellow line. Yellow lines along the long axes of plagioclase microlites are manual length measurements made in ImageJ. Microlite length data are presented in Supplementary Figure 10b.

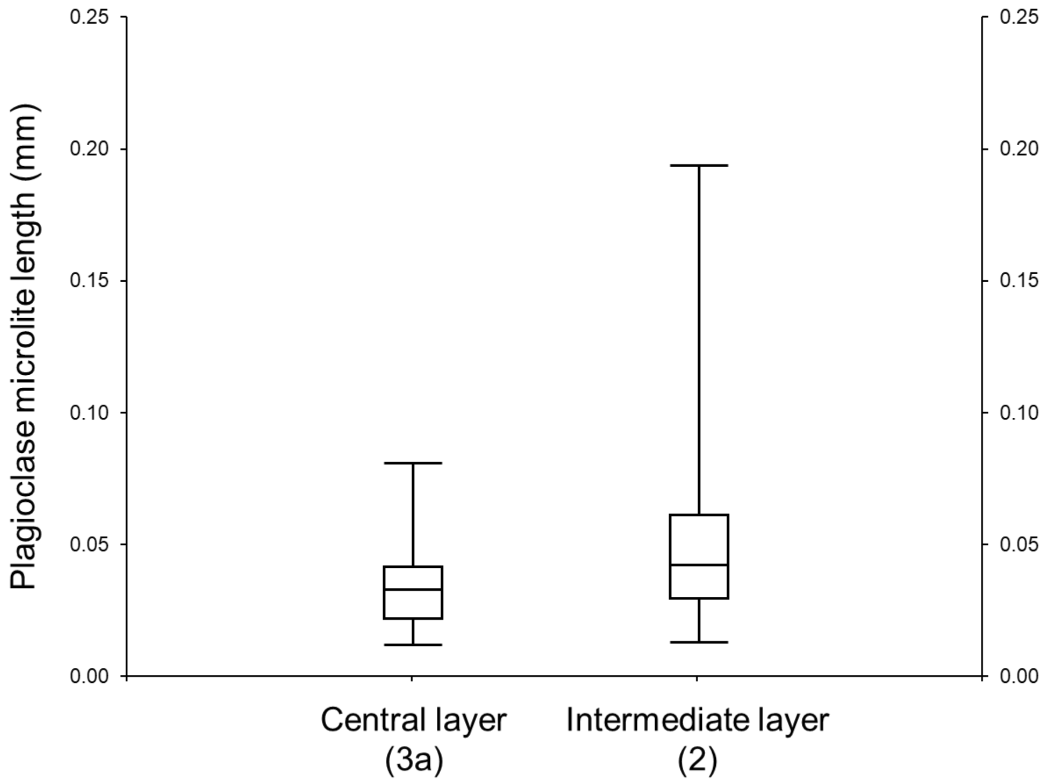

Supplementary Figure 10b. Distribution of plagioclase microlite lengths from either side of the internal chilled margin, in the central and intermediate layers. Outer limits show maximum and minimum measured lengths, whereas the box marks the interquartile range, with the central line showing the median length.
