# Supplementary material for: Magma flow localisation during dyke propagation produces complex magma transport pathways
Source: Nat Commun. 2025 Jul 10;16:6358. doi: 10.1038/s41467-025-61620-5 (PMC12246046; doi:10.1038/s41467-025-61620-5)
Supplement: Supplementary file 2 — Description of Additional Supplementary Files [file 41467_2025_61620_MOESM2_ESM.pdf]

Description of Additional Supplementary Files

File name: Supplementary Data 1
Description: Contains measurements of dyke segment lengths, segment widths and layer widths. Also contains crystal orientation data.
